# Reduced cognitive empathy and differences in EEG activity in low-achieving adolescents

**DOI:** 10.1371/journal.pone.0361520

**Published:** 2026-10-27

**Authors:** Samuel Hernández-González, María Ángeles Gómez-Climent, Albert Clemente-Soriano, Jose Luis Alba Robles, Carlos Caurín-Alonso, Jordi Solbes

**Affiliations:** 1 Department of Experimental and Social Sciences Education, Faculty of Teacher Training, University of Valencia, Valencia, Spain; 2 Legal Area/Faculty of Social Sciences and Law, International University of Valencia, Valencia, Spain; 3 Department of Basic Psychology, Faculty of Psychology, University of Valencia, Valencia, Spain; Federal University of Paraiba, BRAZIL

## Abstract

Students with low academic achievement tend to display more maladaptive behaviours in interactions with peers and teachers. Empathy plays a key mediating role in these interactions and is also linked to academic performance. In particular, facial emotion recognition is one component of socioemotional processing that may contribute to empathic responding. To explore the relationship between empathy, emotion recognition, and academic achievement, a comparative study was conducted with 40 students aged 14–17, divided into two groups: one with low academic achievement enrolled in a school support programme, and a control group with adequate academic performance. A multimethod approach was applied, including the Basic Empathy Scale, a facial expression recognition test, and electroencephalographic (EEG) recordings. Results showed significantly lower cognitive empathy in the low-achieving group in the primary between-group analysis. The overall between-group difference in facial emotion recognition accuracy was not statistically significant. EEG analyses identified exploratory between-group differences in source-level oscillatory activity estimates during facial-expression processing. These electrophysiological findings provide complementary information about task-related activity patterns but should not be interpreted as specific neural markers of empathy or as confirmation of a behavioural facial emotion recognition deficit. Overall, the multimethod approach offers complementary perspectives on socioemotional functioning in low-achieving adolescents, although the exploratory EEG findings require cautious interpretation and replication in larger, independent samples.

## Introduction

Previous research on students who repeat an academic year due to underachievement has identified several common characteristics. In particular, they are at risk of dropping out of school and displaying maladaptive behaviours at a socio-emotional level [1,2].

Spanish secondary schools are legally required to provide “learning and performance improvement groups and the reinforcement programme” (see the article [3] for the specific characteristics of this programme). These programmes are aimed at third and fourth years of compulsory secondary education for students whose poor academic performance prevents them from progressing. Hereafter, we refer to these students as the low academic performance group (LAP).

Some authors [4,5] summarise the characteristics present in these students, which provide a starting point for identifying areas for reinforcement. The authors highlight socio-emotional characteristics that may negatively impact these students’ academic performance, such as poor social skills, emotional instability, and unhealthy social relationships with peers and teachers.

On an emotional level, these students require a more personalised and specific approach to help them develop their emotional intelligence (EI). This will enable them to recognise, regulate, control and express emotions effectively. Working on emotional intelligence has been shown to be an effective strategy for improving academic performance [6].

### Empathy and academic performance

Empathy is a key component of EI that enables one to understand the emotions of others and establish appropriate social relationships. Previous studies have reported positive associations between empathy and academic achievement among students. For example, one of the first studies on this subject found that self-reported empathy in adolescents was related to their grade average or academic achievement [7]. Another study of adolescents considered two components of empathy: empathic concern and perspective-taking. These components contribute to students’ social competence. It was found that both components correlated strongly with social competence, with the latter also correlating with academic performance. These findings suggest that some components of empathy may be associated with academic achievement [8]. Additionally, a sense of coherence (or feeling of trust) and empathy in adolescents are positively associated, and together they are associated with academic achievement [9]. Furthermore, a recent study showed that this relationship with academic performance is mediated by compassion, i.e., feeling empathy and acting to help others accordingly, as well as by commitment to studies [10].

One possible explanation for the relationship between empathy and academic success is that empathetic individuals are sought out for trust and support, occupying central positions in social networks based on trust and closeness [11]. Therefore, an empathic person will be sought out by others and will be able to form supportive and trusting bonds to solve problems, including academic ones. Several studies have shown that empathy can promote prosocial behaviour and facilitate cooperation between members of a group [12]. Interestingly, research shows that prosociality is maintained within a group through reciprocity, either directly (by returning a favour to someone who has helped you previously) or indirectly (by helping those who cooperate with others in general) [13].

In an academic context, a recent study suggests that students who occupy central positions in social networks perform better academically, partly due to positive learning from their social connections [14]. Positive relationships such as these also depend on emotional skills such as empathy. Indeed, students’ levels of empathy play a mediating role in teacher-student relationships and students’ emotional intelligence [15].

In summary, empathy is associated with prosocial skills and may also be relevant to academic functioning—an area that may be lacking in students with low academic achievement. Therefore, the level of empathy in this group of students should be a particular focus of study.

### Empathy and Facial expression recognition (FER) and the brain networks involved

In social cognition, FER is one component of socioemotional processing that may contribute to empathic responding alongside other perceptual, emotional, and social-cognitive processes [16]. Preliminary research has shown a consistent positive relationship between self-reported empathy and FER [17–19]. Additionally, many studies focusing on empathy analysis have used the capacity to recognise emotions through facial expressions as a means to elicit empathy. Most of these studies have focused on evaluating others’ pain, which is considered a form of human empathy. These studies have examined emotion recognition and the modulation of neural activity in areas associated with evaluating emotions in faces [20]. It has been proposed that facial expression recognition involves distributed cortical networks located mainly in occipital, temporal, parietal, and frontal regions that support perceptual face processing and broader socioemotional evaluation [21–33]. Within this framework, some models distinguish between core perceptual systems involved in facial analysis and extended socioemotional systems associated with emotional and social-cognitive processing [21–29]. These distributed networks may contribute to early stages of empathic responding and socioemotional processing. Some cortical regions associated with empathy and socioemotional processing include frontal regions such as the ventromedial (vmPFC), dorsolateral (dlPFC), and dorsomedial prefrontal cortices (dmPFC), as well as the inferior frontal gyrus (IFG); parietal regions including the inferior parietal cortex (IPC), temporo-parietal junction (TPJ), and precuneus/posterior cingulate cortex (PCU/PCC); and temporal regions such as the superior temporal cortex (STC) [34]. In addition, deeper structures such as the anterior insula (AI) and anterior cingulate cortex (ACC) have also been associated with empathy for the pain of others [35].

Within empathy, two partially distinct but interacting dimensions are commonly described: affective empathy and cognitive empathy. Affective empathy refers to the ability to perceive the emotional state of others through observation or imagination [36], whereas cognitive empathy refers to the ability to understand another person’s mental and emotional state and has been linked to Theory of Mind processes [37]. These dimensions may interact depending on contextual and interpersonal variables such as emotional intensity, previous experiences, gender, or social identification with others [38].

The affective pathway has mainly been associated with frontal and temporo-parietal regions, including the anterior insula, anterior medial cingulate cortex, medial frontal cortex, inferior frontal gyrus, and dorsal temporo-parietal junction [39], whereas the cognitive pathway has been related to regions such as the ventral temporo-parietal junction, superior temporal sulcus, and anterior/posterior midline regions [40].

These distributed networks have been implicated in facial emotion recognition and broader aspects of empathic and socioemotional processing [21–30,34,35]. EEG offers a complementary neurophysiological approach for examining task-related activity during emotion processing, although its spatial and methodological limitations require cautious interpretation [41,42]. In the present study, the selected cortical regions were used only as approximate, literature-based reference areas for interpreting exploratory source-level activity estimates obtained during the facial-expression task. Importantly, from a developmental perspective, both empathy and facial emotion recognition (FER) continue to evolve throughout adolescence, reflecting ongoing neurocognitive maturation [43,44]. Evidence indicates that recognition of some emotions, such as fear and disgust, improves markedly during this period, whereas recognition of happiness and sadness follows more gradual trajectories, suggesting differential developmental patterns across emotions. These processes are closely related to the development of cognitive empathy and social cognition. Consequently, differences in FER observed in adolescents may reflect variability in socioemotional developmental pathways rather than fully stabilised abilities [45,46]. Furthermore, recent meta-analytic evidence suggests that the association between empathy and emotion recognition may decrease with age across development, although this effect appears to be modest and the underlying mechanisms remain unclear [47]. In this context, a multimethod approach combining self-report, behavioural and neurophysiological indicators may provide complementary information for characterising socioemotional functioning in adolescents with different academic performance profiles. Previous EEG and neuroimaging studies have shown that differences in neural responses to emotional faces may emerge even when behavioural performance remains comparable across groups, suggesting that neurophysiological measures may provide complementary information about socioemotional processing [48].

### Current study

As previously mentioned, several studies have shown that students with low academic achievement have poor social-emotional skills, and that empathy is related to academic achievement. However, to our knowledge, this is the first study to characterise adolescents in these learning support programmes using self-report questionnaires on empathy and other personal development characteristics, such as social, personal and individualistic values. Despite the existence of articles showing a relationship between the ability to recognise emotions and empathy, none focus on analysing the development of these variables and their relationship in students with low academic performance. Furthermore, no research has investigated empathy in these groups using methodological triangulation (self-report, behavioural, and neurophysiological recording). This multimethod approach may provide complementary information and reduce reliance on any single measure, while acknowledging that the self-report, behavioural, and electrophysiological indicators assess partially distinct aspects of socioemotional functioning [49,50]. Finally, it should be noted that there are also very few studies analysing the role of brain networks associated with these processes in this group of students.

Although previous studies have reported associations between empathy, social competence and academic achievement [7–10,14,15], as well as socioemotional difficulties in students with low academic performance and related learning or behavioural difficulties [4–6,51,52], most research has examined empathy and facial emotion recognition separately. In addition, several studies have shown relationships between empathy and facial emotion recognition [17,18,53–55]. However, the joint analysis of empathy, facial emotion recognition ability, and neurophysiological activity in adolescents with low academic performance remains limited. Examining these variables together may contribute to a more comprehensive understanding of socioemotional functioning in adolescents with different academic performance profiles, and may help to clarify whether observed patterns are associated with variability in socioemotional processing or broader cognitive and contextual factors. This approach may also contribute to clarifying whether socioemotional differences associated with low academic performance reflect differences in socioemotional processing related to facial emotion recognition and empathy, or secondary effects linked to broader cognitive, motivational, or contextual factors. Combining behavioural and neurophysiological indicators may help characterise whether group differences are more evident at the behavioural or electrophysiological level, without implying a specific neural mechanism. Although EEG does not directly measure activity in deep subcortical structures, power spectral density (PSD) analyses combined with source estimation provide exploratory information about task-related cortical activity patterns. Given the spatial limitations of the EEG configuration, these estimates cannot establish precise anatomical localisation or determine whether the observed activity is specific to facial emotion recognition or empathy. Recent reviews highlight both the growing contribution of EEG methods to emotion research and the methodological challenges involved in interpreting EEG-based emotion-recognition findings [41,42]. The objective of this study was to ascertain the level of empathy in the target group and its potential educational implications. In order to achieve this, the following questions were raised:

The present study seeks to contribute to the extant literature by investigating whether the LAP group will be characterised by low levels of personal and social value development, as has been posited in other studies.

The primary research question guiding this study is whether the LAP group will show a significantly lower level of empathy development in comparison to a control group.

The EEG analyses were intended to explore between-group differences in oscillatory activity during facial-expression processing and to provide complementary electrophysiological information about task performance.

Finally, we examined whether empathy and facial emotion recognition performance were associated with exploratory source-level oscillatory activity estimates obtained during the facial-expression task. Based on the reviewed studies (see the “Empathy and Academic Achievement” section) and the research questions, we hypothesise that the LAP group will exhibit poor social value development and show significantly lower empathy than the control group. We further hypothesise that the LAP group will show lower facial emotion recognition performance than the control group. We also explored whether the groups differed in oscillatory activity across conventional EEG frequency bands during facial-expression processing. Given the exploratory nature of these analyses, no interpretation specific to empathy or to a single neural system was assumed. Together, these measures were intended to provide complementary information about socioemotional and task-related functioning in adolescents with different academic performance profiles.

## Methods

A multimethod approach was used to study empathy in these groups of adolescents. First, empathy was assessed using a self-report questionnaire. Second, a behavioural approach was employed to evaluate the ability to recognise facial emotional expressions. Finally, an electrophysiological analysis was conducted using EEG activity recorded during the facial-expression recognition task.

### Statement of ethical approval

This study was conducted in accordance with the Declaration of Helsinki and was approved by the Ethics Committee of the University of Valencia. All subjects and/or their tutors gave written informed consent prior to participation for the publication of information relating to the test results. In addition, the study did not publish any data or images that would lead to the identification of the children, and data files were named under the pseudonymisation protocols established by the local Ethics Committee of the University of Valencia.

### Participants

We recruited 40 participants from a secondary school. The vast majority of students in this school were from a middle-class background. The sample included a very low percentage of students of foreign origin. The ages of the participants ranged from 14 to 17.

A first group of participants (20 participants) consisted of adolescents belonging to the institutional reinforcement programme, which includes students who are not able to progress to the next grade because of their low academic performance (LAP group). A second group was composed of 20 students who had passed all their subjects (control group).

Due to data quality criteria applied to EEG recordings, one participant was excluded from the neurophysiological analyses, resulting in a final EEG sample of 39 participants (LAP: n = 20; control: n = 19). Self-report and behavioural analyses were conducted using the full sample where data were available.

The sample size is consistent with those commonly reported in EEG studies of emotional processing [42,56,57]. A sensitivity power analysis indicated that the final EEG sample size was sufficient to detect large effect sizes (Cohen’s d ≈ 0.92), although smaller effects may have remained undetected.

All participants had no history of neurological or psychiatric impairment and had normal (or corrected-to-normal) vision. They were right-handed, as assessed using a Spanish abridged version of the Edinburgh Handedness Inventory [58]. Students from both the reinforcement programme (LAP group) and conventional classrooms (control group) were informed about the general characteristics of the study without revealing the experimental grouping, and participation was voluntary.

### Procedure and instruments

The study combined self-report, behavioural, and neurophysiological measures to assess empathy and related socioemotional processes in the two groups of students.

#### Basic empathy, social skills, and values in adolescents scales.

Firstly, we assessed the development of social skills and values in the two groups of adolescents employing the Social Skills Scale and the Values in Adolescents Scale [59] to verify the psychosocial characterisation previously carried out by other authors in students with low academic performance and belonging to the learning enhancement programme groups. The first scale contains a total of 12 items to assess the factors: “communication skills”, “assertiveness”, and “conflict resolution” by means of a seven-level Likert scale ranging from “absolutely false” to “absolutely true”. The second scale (Values in Adolescents Scale) contains a total of 24 items to assess the factors: social values (consisting of the second-level factors “justice and equality,” “pro-sociality” and “commitment”), personal values (“honesty,” “responsibility” and “integrity”) and individualistic values (“hedonism” and “social recognition”) using a seven-level Likert scale ranging from “not important” to “the most important”.

To analyse the self-reported empathy, we used the adaptation and translation into Spanish of the Basic Empathy Scale included in Oliva et al. [59], which allows independent evaluation of affective and cognitive empathy, in addition to global empathy. It is composed of nine items, four assessing affective empathy and five assessing cognitive empathy, to be scored on a five-level Likert scale ranging from “strongly disagree” to “strongly agree”.

The students completed these questionnaires before the behavioural test and the electroencephalographic recording. Each questionnaire was coded to identify it within one of the two experimental groups (control or LAP).

#### Facial expression recognition test.

In the domain of social cognition, facial expression recognition (FER) is one component of socioemotional processing that may contribute to empathic responding alongside other perceptual, emotional, and social-cognitive processes [16–18]. Specifically, some studies have shown a positive correlation between self-reported questionnaires to measure empathy components, such as the Interpersonal Reactivity Index (IRI test), and tests of facial expression recognition and pain assessment in others [53]. Another similar study focused on evoked responses during facial-expression recognition and examined correlations between trait empathy, measured using the IRI, and facial expression recognition, yielding analogous results [54]. In addition, even though empathy involves both the affective and cognitive components [60], classifying emotions without evaluating their valence (positive or negative, pleasurable or unpleasurable, calm or excited, etc.), like when categorising facial expressions, seems to preferentially trigger the cognitive component [61,62].

In this test, our participants performed a facial expression recognition (FER) task, in which they had to identify a facial expression from among seven possible ones. For this purpose, we used a set of pictures of human faces of 70 individuals, the Karolinska Directed Emotional Faces (KDEF) [63], displaying 7 basic facial expressions: fear, anger, disgust, happiness, sadness, surprise and neutral. Fifty-six images containing the faces of 56 different individuals, displaying one of the seven expressions, were selected. An equal distribution of expressions was ensured in this set of 56 images (8 images for each expression). The alphabetical order of the name of the KDEF images was used to make the selection. Each participant evaluated the set of 56 images. The order of presentation of the set of 56 images was automatically randomised without replacement by means of the option contained in the E-Prime application (RRID:SCR_009567; Psychology Software Tools, Inc., Sharpsburg, Pennsylvania, USA).

This test was performed in a sound-attenuated room, where participants sat comfortably, without the presence of other people, in a darkened environment and with no visual stimuli other than from the experimental setting. These images were presented on a high resolution monitor approximately positioned at eye level, 80 cm away and in front of the participants. Before starting the test, each participant was informed about the procedure. Moreover, a series of on-screen prompts informed about the steps they had to take before the test. Each trial started with a fixation image, a white “ + ” symbol on a black background, which lasted two seconds (Fig 1A; “fixation period”). After these two seconds, this symbol was replaced with the image of a facial expression (stimulus). This facial expression was kept on the screen for 6 seconds (Fig 1A; ‘stimulus period’). Finally, an evaluation method appeared under this image (Fig 1A, ‘evaluation period’), consisting of seven buttons indicating an emotion and allowing the participant to evaluate the facial expression of the image. For this period, no time limit was established in order to know the reaction time of each response. No time interval was used between the 56 trials. This test was created using the E-Studio software (E-Prime; RRID:SCR_009567; Psychology Software Tools, Inc., Sharpsburg, Pennsylvania, USA).

**Fig 1:**
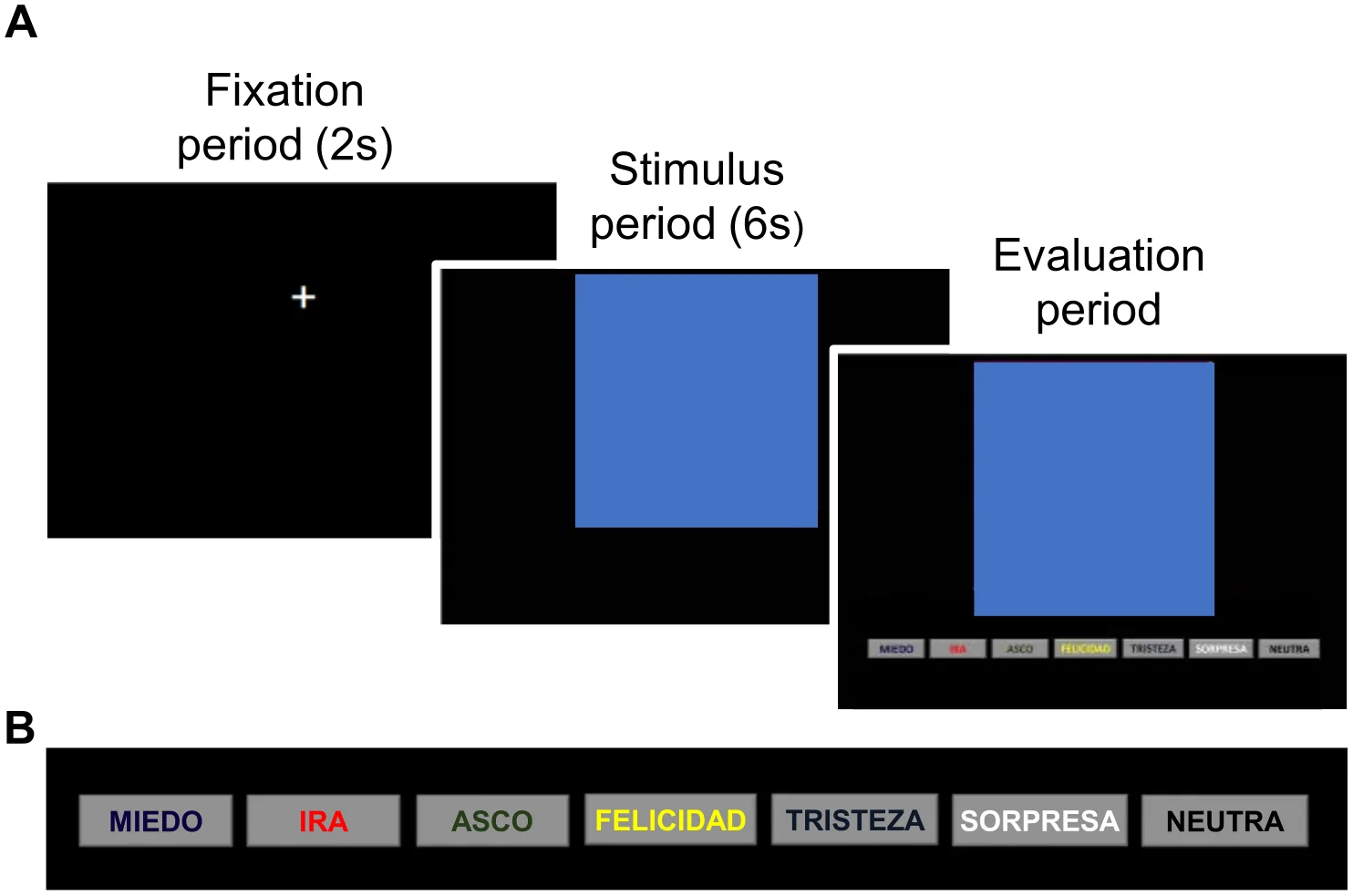
Experimental design. A. Sequence of events displayed during the tests of facial emotion recognition. Blue rectangles indicate the position where KDEF facial stimuli would normally appear, omitted here due to copyright restrictions. B. Magnification of the lower portion of the image on the right in 1A (evaluation period), showing in detail the evaluation method for the facial expression recognition test. Buttons indicate an emotion; from left to right: fear, anger, disgust, happiness, sadness, surprise and neutral.

#### Electroencephalographic activity.

During the FER test, the electroencephalographic activity of the participants was recorded. Our analysis focused on EEG activity recorded during the presentation of emotional facial expressions.

We also used the electroencephalographic activity of the fixation period, which was considered a visual stimulus devoid of emotional content, to calculate the oscillatory activity elicited by a non-emotional stimulus. The fixation period provided a non-emotional visual comparison condition for interpreting task-related oscillatory activity.

In addition, the resting-state activity of each participant was recorded for 180 seconds, just after the impedance levels of the different electrodes were checked and before the start of the tests. During this period, in which there was an absence of explicit stimuli, participants were asked to remain calm, with their eyes open and looking straight ahead and, as far as possible, avoiding movement.

Participants were asked to minimise blinking and eye movements to minimise subject-generated artefacts in the EEG signal during the period of visualisation of the images of the FER test.

### Recordings

#### Basic empathy scale and values in adolescents scale.

The participants’ responses for these scales were obtained on paper and transferred to an Excel spreadsheet (v. 2307). Responses to the Basic Empathy Scale were coded from 1 (“strongly disagree”) to 5 (“strongly agree”), whereas responses to the Values in Adolescents Scale were coded from 1 (“not important”) to 7 (“the most important”).

#### Emotional images assessment tests.

The E-Prime software E-DataAid was used to record all responses in the facial expression recognition test (E-Prime; RRID:SCR_009567; Psychology Software Tools, Inc., Sharpsburg, Pennsylvania, USA). Correct responses were transformed into the value ‘1’ and the incorrect ones into the value ‘0.’ In addition to the values corresponding to the evaluation of FER, the reaction time was measured.

#### Electroencephalographic activity.

The continuous electroencephalographic (EEG) activity was recorded during the FER test using an elastic cap (EASYCAP GmbH; Herrsching, Germany) provided with 24 electrodes Ag/AgCl that were positioned according to the 10/20 International System (Fp1, Fp2, F3, F4, C3, C4, P3, P4, O1, O2, F7, F8, T7, T8, P7, P8, Fz, Cz, Pz, M1, M2, AFz, CPz, POz) covering scalp regions associated with cortical networks of interest in the present study. The montage provided broad coverage of frontal, central, temporal, parietal, and occipital scalp regions. These broad scalp territories were selected to provide coverage relevant to the distributed cortical systems involved in face processing described in previous literature [64]. However, scalp electrode positions should not be interpreted as providing direct correspondence with specific cortical structures. The ground electrode was attached to the AFz position, in the middle frontal area, and the left mastoid bone (M1) was used as the reference. Electrode impedance was monitored so that it was generally below 5 kΩ and always below 10 kΩ. The EEG activity was acquired at a sampling rate of 500 Hz by a mBrainTrain Smarting amplifier (RRID: SCR_017634; mBrainTrain LLC, Belgrade, Serbia). This amplifier is a 24-channel wireless EEG device (22 recording channels plus 1 reference and 1 ground) with 24-bit resolution, input impedance of 1 GΩ and input referred noise < 1 µV. No dedicated EOG channels were recorded. To minimise ocular artefacts, participants were instructed to avoid excessive blinking and eye movements during stimulus presentation, and ocular activity was subsequently attenuated using SSP-based artefact correction and visual inspection procedures.

### Analysis

#### Electrophysiological signal analysis.

To analyse the EEG data, we used the Brainstorm software (version 3.19111, USC & McGill University, California, USA; RRID: SCR_001761) based on the Matlab programming tool (v.R2018a, Mathwork, RRID: SCR_005547). First, we preprocessed the signal with an offline 50 Hz notch filter, a 0.1 Hz high-pass filter, and a 110 Hz low-pass filter. EEG artefacts were corrected using Signal Space Projection (SSP) implemented in Brainstorm. Specifically, SSP was applied to attenuate ocular activity (eye blinks and saccadic eye movements), cardiac activity, and movement-related artefacts while minimising distortion of the underlying neural signal. Artefact-related events used for SSP decomposition were identified through semi-automatic detection procedures implemented in Brainstorm, based on amplitude and waveform characteristics of ocular, cardiac, and movement-related activity patterns. Detected events were subsequently visually inspected before SSP computation. This approach has been reported to provide effective attenuation of stereotyped EEG artefacts while maintaining a simpler implementation compared with ICA-based procedures [65]. For each detected event, a short time window around each of these event markers was extracted and concatenated over time. In a second stage, we proceeded to the removal of artefacts. In this process, a principal components analysis (PCA) was applied and the components to remove were selected. A linear projector was computed for each spatial component and it was applied to remove the artefact contributions. In our case, two components were selected, which presented significantly higher contributions to the artefacts of interest than the rest of the components. After this selection, the records were checked to confirm the correct removal of the artefacts. This procedure was repeated for each artefact type and was applied to each of the participants’ records. Residual artefact-contaminated epochs were additionally identified through visual inspection and excluded from subsequent analyses when necessary. Specifically, epochs containing more than one eye blink during the 2 s interval following stimulus presentation, as well as segments showing prolonged muscular contamination or signal saturation exceeding 250 ms, were rejected. In practice, these artefact-contaminated segments typically exceeded amplitudes of ±100 μV. The proportion of rejected epochs remained relatively low and comparable between groups, with a mean rejection rate of 12.59% (SD = 8.17) in the control group and 12.32% (SD = 7.57) in the LAP group. Additional preprocessing quality-control analyses examined pre- to post-SSP amplitude changes, frontal EEG proxy indicators of ocular-related activity, residual post-SSP amplitude distributions, and representative pre-/post-SSP waveforms. These analyses showed that SSP-related amplitude changes were predominantly concentrated over anterior electrodes, whereas epoch rejection rates and residual post-SSP amplitudes over non-frontal channels were broadly comparable between groups. This pattern supports the broad comparability of the EEG data retained for the subsequent source-level PSD analyses, although the evidence regarding ocular-artefact attenuation remains indirect because dedicated EOG channels were not recorded (detailed procedures and complete quality-control results are provided in S1 Text, S1–S3 Tables, and S1 Fig.).

These preprocessed data were used to estimate source-level cortical activity. The volume conductor model used a symmetric boundary element method (symmetric BEM), which employs three realistic layers: scalp, inner skull and outer skull. The relative conductivities were set to 1, 0.0125 and 1, respectively. The amplitude of brain electrical currents distributed over a previously calculated cortical surface model (15002 vertices) was estimated using the minimum norm imaging method. The sLORETA variant was used with the unconstrained option for the dipole orientation [66]. The calculation of noise covariance and data covariance matrices was required. For the calculation of these matrices, the resting-state data and the fixation periods were used accordingly. These matrices were calculated for each participant.

Then, we calculated the power spectral density (PSD) [67]. This process involved dividing signals into overlapping windows with a specified length, computing the Fast Fourier Transform (FFT) of each short segment, and averaging the power of the FFT coefficients across all overlapping windows. This method has been widely employed in numerous studies to examine electroencephalographic responses to emotional stimuli, as well as in investigations on epilepsy and the impact of transcranial direct current stimulation [68–71] for comparing differences between the typically utilised frequency bands in EEG analysis.

The PSD was calculated for each of the trials of the participants in both groups. This was carried out for the fixation period and the stimulus period (Fig 1A). In the case of the calculation of the PSD in the stimulus period, it was computed for the seven facial expressions evaluated in the FER test. The time window for calculating the PSD was 2 seconds from stimulus onset (the facial expression image), covering the stimulus response times in facial expression recognition proposed in the literature [24,72]. PSD was also calculated for the fixation period, for which the 2-second period was selected from the appearance of the fixation feature (a white cross on a black background). A 1-second window length with a 50% overlap ratio was employed. A Hamming window was applied.

This PSD per trial was subsequently used to calculate the statistical differences between the control and LAP groups for each facial expression. For each facial expression condition, the EEG analyses included eight trials per participant, resulting in 160 trials for the LAP group and 152 trials for the control group after exclusion of one control participant due to EEG data quality criteria. This analysis was performed for the most commonly used frequency bands (theta: 4–7 Hz, alpha: 8–12 Hz, beta: 13–30 Hz, low gamma: 31–50 Hz and high gamma: 51–100 Hz). Given the exploratory nature of the present study, analyses were conducted across conventional EEG frequency bands commonly examined in emotional processing research, rather than focusing on specific a priori hypotheses regarding individual oscillatory bands. Previous studies have reported changes in theta-, alpha-, beta-, and gamma-band activity during facial-expression processing, although the functional interpretation of these oscillations varies across experimental paradigms. Theta oscillations have been associated with early structural and emotionally salient face processing within occipito-temporal regions [73], whereas alpha activity has been linked to attentional regulation and inhibitory control during social perception and structural facial encoding [73,74]. Beta-band oscillations have been associated with higher-order socio-cognitive processing, sensorimotor simulation, and interregional coordination during face-processing tasks, including activity recorded over somatosensory, temporo-parietal, and prefrontal regions [73,75]. Gamma-band activity has been related to perceptual binding and the processing of emotionally salient facial information involving temporal and prefrontal regions [75]. Previous EEG studies suggest that these oscillatory dynamics may involve distributed perceptual and socioemotional systems engaged during facial-expression processing [73,75]. Recent EEG-based emotion classification studies have identified beta- and gamma-band activity as potentially informative features for distinguishing emotional states [75]. However, the functional meaning of these frequency bands depends on the task, recording procedures, and analytical approach; therefore, they should not be interpreted in the present study as process-specific neural markers of facial emotion recognition or empathy.

#### Statistical analysis.

Behavioural, psychosocial and correlational analyses were performed using Sigmaplot (v.11, Systat Software, Inc., Germany; RRID:SCR_003210) and Jamovi software (v.2.7.24, Sydney, Australia; RRID:SCR_016142). In these tests, population samples were first tested for normality using the Shapiro–Wilk test. For psychosocial and emotional scales, the control and LAP groups were compared using the parametric Student’s t-test or the non-parametric Mann–Whitney test, depending on whether the assumption of normality was met.

For the FER task, behavioural performance was analysed using mixed-design repeated-measures ANOVAs with group (control vs. LAP) as a between-subjects factor and facial-expression category (fear, anger, disgust, happiness, sadness, surprise, and neutral) as a within-subject factor. Recognition accuracy and reaction time were analysed separately as dependent variables. A significance level of α = 0.05 was adopted for behavioural and psychosocial analyses.

Statistical differences in electroencephalographic activity between groups were calculated for the fixation period and the stimulus period of every facial expression. The fixation and facial-expression conditions were analysed separately; fixation-related contours were used only as a descriptive spatial reference, and no direct fixation-versus-expression statistical contrast was performed.

Between-group differences in PSD were analysed using the statistical tools implemented in Brainstorm software. The non-parametric permutation-based Student’s t-test was employed because the data of the control and LAP groups deviated from the normal distribution. A total of 10,000 randomisations or permutations between the data from the control and LAP groups were used to determine non-random differences between the control and LAP groups. A significance threshold of α = 0.001 was adopted for EEG analysis. EEG maps were thresholded using the empirical two-sided permutation p values at p < .001. The corresponding boundary value in the permutation output was |t| = 2.5127. To control for multiple comparisons, a false discovery rate (FDR) correction was applied using the same Benjamini–Hochberg procedure [76] across all analyses. In EEG analyses, the correction was implemented across time, frequency, and spatial dimensions (signals/vertices). Benjamini–Hochberg FDR correction was applied separately to four prespecified families: 18 psychosocial comparisons, five age- and sex-adjusted group effects, three FER–empathy correlations, and 60 EEG–behaviour correlations. FDR was recalculated within the corresponding family in each sensitivity-analysis iteration. FER behavioural analyses controlled for multiple comparisons using Tukey’s HSD post hoc procedure. Effect sizes were reported for all statistical tests (Cohen’s d for t-tests, rank-biserial r for Mann–Whitney tests, partial eta squared (η^2^p) for ANOVA models, and Spearman’s rho (ρ) for correlation analyses).

Supplementary sensitivity analyses were conducted to assess the robustness of the results that met the prespecified statistical criterion. These analyses examined the influence of observations flagged by a prespecified potential-outlier rule and of omitting participants one at a time. Age- and sex-adjusted models were additionally fitted for the focal psychosocial comparisons. All observations were retained in the primary analyses, and exclusions were applied only within the corresponding sensitivity analyses. Detailed procedures and complete results are provided in S2 Text. EEG robustness was examined using candidate-outlier exclusions within the original trial-level framework and participant-level leave-one-out analyses across 39 participants, seven conditions, and three frequency bands (S3 Text).

Associations between FER performance and empathy measures were analysed in the full behavioural sample (n = 40). Associations involving EEG-derived PSD measures were analysed in participants for whom complete EEG, behavioural, and psychosocial data were available (n = 39). Partial correlation analyses controlling for age, sex, and group membership were performed. Spearman partial correlations were used due to the absence of normality in the analysed variables. Correlations were calculated between FER performance, empathy measures, psychosocial variables, and PSD values extracted from five literature-based cortical reference regions located within broad posterior temporal, occipital, ventral occipito-temporal, anterior temporal, and somatosensory territories. Twelve vertices were selected for each reference region based on previously published anatomical descriptions [64], and the same vertices were used across participants and facial-expression conditions. For each participant, facial-expression condition, and frequency band, the 12 vertex-level PSD values were averaged to obtain a single regional value. Given the limited spatial resolution of the 22-channel EEG recording configuration, these labels were used only to define reproducible sampling locations and should not be interpreted as precise anatomical localisations. Frontal and prefrontal regions were not included among the regional sampling locations used for the correlational analyses because, in the absence of dedicated EOG channels, signals in these areas are particularly susceptible to residual ocular and periocular muscular activity. Frontal clusters visible in the whole-cortex statistical maps were retained for completeness but were not subjected to region-specific functional interpretation.

In the following section, the statistical tests used in each study are given in parentheses, together with the U, t, or F statistics, corrected p-values, confidence intervals, effect sizes, and, where applicable, 95% confidence intervals for Cohen’s d estimates.

## Results

### Empathy, social skills and values in adolescents

After FDR correction, significant between-group differences were found for cognitive empathy, social values, justice and equality, personal values, and honesty (Table 1). For each of these variables, the control group obtained higher scores than the LAP group. Global empathy, affective empathy, social skills, communication skills, assertiveness, conflict resolution, prosociality, commitment, integrity, responsibility, individualistic values, social recognition, and hedonism did not differ significantly between groups after FDR correction.

**Table 1:** Between-group comparisons for empathy, social skills, and values. The value in parentheses following Cronbach’s alpha indicates the number of items included in each scale or subscale. Significant FDR-adjusted p values are shown in bold. Ninety-five per cent confidence intervals are reported only for Cohen’s d effect sizes derived from Student’s t-tests, as confidence intervals for rank-biserial correlations were not estimated in the Mann–Whitney U analyses.

Variable	Component	Cronbach’s alpha	Test statistic	FDR-adjusted p value	Effect size	95% CI
Empathy		0.77 (9)	t = 2.069	0.076	d = 0.788	[0.279, 1.625]
	Cognitive	0.73 (5)	U = 90.00	**0.026**	r = 0.550	—
	Affective	0.78 (4)	t = 0.512	0.703	d = 0.162	[-0.346, 0.933]
Social Skills		0.64 (12)	t = 2.275	0.068	d = 0.579	[-0.074, 1.225]
	Communication skills	0.44 (5)	t = 2.202	0.068	d = 0.176	[-0.463, 0.812]
	Assertiveness	0.68 (3)	t = 1.106	0.417	d = 0.515	[-0.136, 1.158]
	Conflict resolution	0.75 (4)	U = 115.50	0.162	r = 0.360	—
Social Values		0.77 (9)	U = 89.00	**0.028**	r = 0.507	—
	Justice and equality	0.74 (3)	U = 43.00	**0.018**	r = 0.762	—
	Prosociality	0.72 (3)	t = 2.205	0.068	d = 0.715	[0.054, 1.367]
	Commitment	0.65 (3)	U = 169.00	0.790	r = 0.064	—
Personal Values		0.66 (9)	U = 87.50	**0.028**	r = 0.515	—
	Honesty	0.63 (3)	U = 89.50	**0.028**	r = 0.504	—
	Integrity	0.72 (3)	t = 2.502	0.051	d = 0.812	[0.144, 1.469]
	Responsibility	0.43 (3)	t = 0.493	0.703	d = 0.160	[-0.478, 0.796]
Individualistic Values		0.76 (6)	t = 0.683	0.642	d = 0.228	[-0.411, 0.865]
	Social recognition	0.85 (3)	t = 0.146	0.885	d = 0.059	[-0.577, 0.695]
	Hedonism	0.77 (3)	t = 1.049	0.417	d = 0.340	[-0.303, 0.979]

Supplementary sensitivity analyses showed that all five focal between-group differences retained their original direction and remained significant after FDR adjustment following exclusion of observations identified by the prespecified potential-outlier rule. All five findings also remained significant in each of the 40 participant-level leave-one-out iterations, with no effect-direction reversals. After adjustment for age and sex, the differences in justice and equality, honesty, social values, and personal values remained significant after FDR correction, whereas the cognitive-empathy group coefficient was attenuated and its confidence interval included zero (S2 Text, Tables A–C and Fig A).

### Behavioural

Behavioural performance in the FER task was analysed using mixed-design repeated-measures ANOVAs, with group as a between-subjects factor and facial-expression category as a within-subject factor. For recognition accuracy, neither the main effect of group (F(1, 38) = 3.252, p = 0.079, η^2^p = 0.079) nor the group-by-emotion interaction (F(6, 228) = 1.316, p = 0.251, η^2^p = 0.033) was statistically significant. For reaction time, neither the main effect of group (F(1, 38) = 0.373, p = 0.545, η^2^p = 0.010) nor the group-by-emotion interaction (F(6, 228) = 0.648, p = 0.692, η^2^p = 0.017) was statistically significant.

A significant main effect of facial-expression category was observed for recognition accuracy (F(6, 228) = 61.855, p < 0.001, η^2^p = 0.619) and reaction time (F(6, 228) = 9.347, p < 0.001, η^2^p = 0.197), indicating that performance varied across emotion categories independently of group membership. Descriptive means for each emotion category are presented in Fig 2.

**Fig 2:**
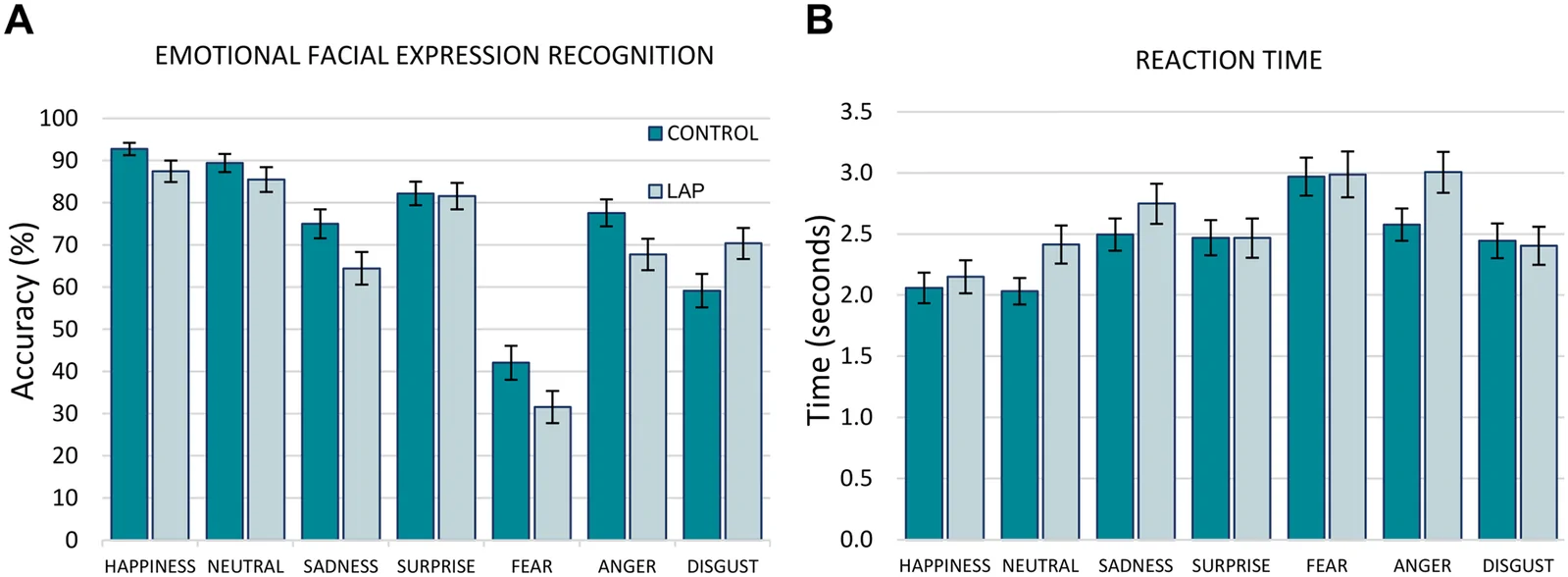
Evaluation of emotions in facial expressions. **a.** Percentage of correct responses of each of the two groups (control and LAP) for each of the seven facial expressions. **b.** Reaction times for facial emotion recognition.

### Electrophysiological

Exploratory source-level analyses identified between-group differences meeting the prespecified statistical threshold during both the fixation and facial-expression conditions (Fig 3F). The interpretation of the statistical results for the facial-expression conditions focused on regions outside the areas that also showed between-group differences during fixation.

**Fig 3:**
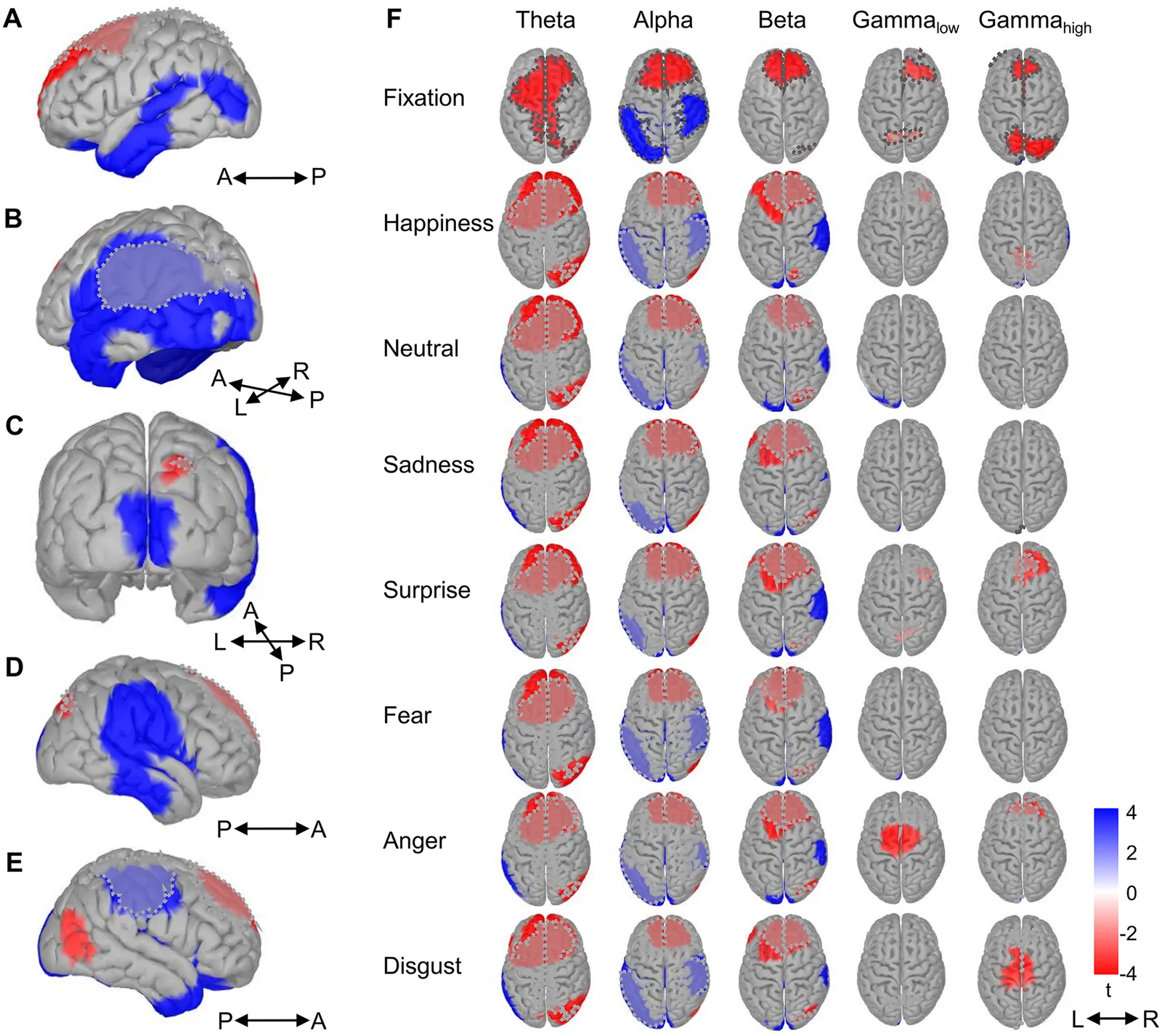
Comparative analysis of approximate source-level oscillatory activity estimates between groups during facial-expression processing. Clusters meeting the prespecified statistical threshold are shown in red and blue. Blue areas indicate lower spectral power in the LAP group, whereas red areas indicate higher spectral power in the LAP group relative to the control group. A–E show selected illustrative examples of between-group differences within broad cortical territories. F shows the complete representation across facial-expression conditions and frequency bands. Frontal clusters visible in the whole-cortex maps are shown for completeness but are not interpreted because of their greater susceptibility to residual ocular and periocular muscular activity. Dashed grey contours indicate fixation-related between-group differences and are shown only as a descriptive spatial reference. A: anterior; P: posterior; L: left; R: right.

Selected differences meeting the statistical threshold included lower theta- and alpha-band activity in the LAP group within broad left temporal and occipital regions, lower beta-band activity within broad right temporal and parietal regions, and lower activity within broad bilateral anterior temporal regions. Increased alpha-band activity in the LAP group was also observed within a broad right temporo-occipital region. The complete condition-wise results are presented in Fig 3F.

These between-group differences met the statistical empirical permutation threshold (two-sided p < .001; corresponding |t| ≥ 2.5127) but their spatial and functional interpretation remains exploratory. The spatial descriptions were informed by previous literature on broad cortical territories associated with facial-emotion processing [22,77] and should not be interpreted as precise source localisations.

Supplementary candidate-outlier and participant-level leave-one-out analyses indicated that the EEG topographies were broadly stable. In the leave-one-out analysis, 813 of 819 recalculated maps showed neither a spatial correlation below .90 nor sign agreement below 80%. The main sensitivity was concentrated in the Fear condition, particularly after omission of CONTROL-01 (S3 Text; S2–S3 Figs).

Partial Spearman correlations controlling for group, age, and sex showed that FER performance was positively associated with cognitive empathy after FDR correction (ρ = 0.411, p = .0114, FDR-adjusted p = 0.034). FER performance was not significantly associated with global empathy (ρ = 0.112, FDR-adjusted p = 0.508) or affective empathy (ρ = −0.008, FDR-adjusted p = 0.960).

The positive FER–cognitive empathy association retained its direction in the supplementary sensitivity analyses but showed limited stability of statistical significance. After exclusion of the five observations identified by the potential-outlier rule, the association was attenuated and no longer significant after FDR adjustment (partial Spearman ρ = .331, FDR-adjusted p = .167). In the leave-one-out analysis, the correlation remained positive in all 40 iterations, although FDR-adjusted significance was retained in 31 iterations (77.5%). Thus, the direction and approximate magnitude of the association were relatively stable, but its statistical significance depended partly on individual observations (S2 Text, Tables D–E and Fig B).

None of the associations between oscillatory activity and FER or empathy-related measures remained statistically significant after FDR correction (Table 2). No statistically significant associations were observed between FER or empathy-related measures and the remaining socioemotional variables after controlling for group, age, and sex and applying FDR correction.

**Table 2:** Partial Spearman correlations controlling for age, sex, and group membership. Correlation coefficients are presented descriptively as effect-size estimates. None of the associations shown in this table remained statistically significant after FDR correction; therefore, no pre-correction significance markers are displayed. Row labels identify broad, literature-based cortical reference regions used for PSD extraction and do not imply precise anatomical source localisation. FER: facial emotion recognition. N = 39.

	FER			Empathy			Affective			Cognitive		
	Theta	Alpha	Beta	Theta	Alpha	Beta	Theta	Alpha	Beta	Theta	Alpha	Beta
Posterior temporal	−0.200	−0.145	−0.037	−0.041	−0.024	0.334	0.181	0.129	0.327	−0.310	−0.258	0.181
Occipital	−0.208	0.023	0.074	−0.148	0.025	0.370	0.023	0.050	0.274	−0.292	−0.077	0.277
Ventral occipito-temporal	−0.168	0.022	0.015	−0.168	0.058	0.379	0.043	0.113	0.309	−0.358	−0.120	0.303
Anterior temporal	−0.214	−0.170	−0.108	0.042	0.179	0.326	0.200	0.225	0.316	−0.231	−0.045	0.242
Somatosensory	−0.305	−0.215	−0.003	−0.075	−0.031	0.358	0.210	0.128	0.380	−0.395	−0.264	0.231

## Discussion

The present study identified significantly lower cognitive empathy in the LAP group, together with exploratory between-group differences in source-level EEG oscillatory activity during facial-expression processing. The overall group effect on facial emotion recognition accuracy was not statistically significant. Accordingly, the self-report, behavioural, and electrophysiological results should be considered complementary observations rather than convergent evidence of a single socioemotional or neural deficit. Although the EEG differences met the prespecified statistical threshold, their anatomical and functional interpretation remains exploratory. The EEG findings do not establish a neural basis for reduced cognitive empathy and require replication in larger samples.

### Self-reported questionnaires and behavioural results

Adolescents with low academic performance who participated in learning enhancement programmes showed significantly lower cognitive empathy, social values, justice and equality, personal values, and honesty than the control group. Global empathy and affective empathy did not differ significantly between groups after FDR correction. These findings are consistent with previous studies that examined social and socioemotional functioning in this group of students using interviews and qualitative methods [4,5]. The significant between-group difference in empathy was therefore limited to the cognitive component assessed by the Basic Empathy Scale.

The sensitivity analyses strengthened the evidence for the four values-related group differences, which remained stable after potential-outlier exclusion, leave-one-out analyses, and adjustment for age and sex. Cognitive empathy showed a more qualified pattern. The LAP group was significantly older than the control group (M = 16.09, SD = 1.06 vs. M = 14.81, SD = 0.46 years; Welch’s p < .001; S2 Text, Table C, Panel A), and the cognitive-empathy group coefficient was attenuated by 47.3% and was no longer statistically significant after adjustment for age and sex (S2 Text, Table C, Panels B–C). However, age was not an independent significant predictor in the full model, and the within-group associations between age and cognitive empathy were not significant. Thus, the sensitivity analysis indicates substantial demographic overlap between age and group rather than demonstrating that age caused the original difference. Moreover, this pattern is not readily explained by a simple normative age-related decline between 14 and 17 years, as reviews of longitudinal and cross-sectional evidence generally indicate continued, although heterogeneous and sex-dependent, development of perspective-taking abilities across adolescence [78,79]. The lower cognitive empathy observed in the older LAP group may therefore also reflect other psychosocial or contextual characteristics associated with this educational profile, such as differences in interpersonal experiences or the broader socioemotional environment, although these possibilities were not directly tested. Accordingly, the adjusted result warrants caution and does not establish whether age or other group-related factors accounted for the original difference.

The overall group effect on facial emotion recognition accuracy was not statistically significant; therefore, the present study does not provide evidence of a behavioural FER impairment in the LAP group. Accordingly, the hypothesis that the LAP group would show lower FER performance was not supported. FER performance was, however, positively associated with cognitive empathy after controlling for group, age, and sex and applying FDR correction. The supplementary analyses qualified this result. The association retained a positive direction after exclusion of observations identified as potential outliers and in all participant-level leave-one-out iterations, but it no longer remained significant after the potential-outlier exclusion and retained FDR-adjusted significance in only 77.5% of the leave-one-out iterations. The consistency of its direction suggests that the association was not produced by a simple reversal driven by one participant; nevertheless, its statistical significance was moderately sensitive to influential observations and should be regarded as preliminary rather than as a robust confirmatory finding (S2 Text, Tables D–E and Fig B). This result suggests that the two measures may capture related but partially distinct aspects of socioemotional functioning and is consistent with previous studies reporting associations between facial-expression recognition and cognitive empathy [17,18,53–55]. Previous research has suggested that empathy and broader socioemotional functioning may be associated with students’ position within classroom social networks, their relationships with peers and teachers, and the support available to them [11,14,15]. Such social and contextual factors may contribute to difficulties associated with academic performance, although they were not directly assessed in the present study. Future studies should therefore examine whether these variables contribute to the associations among cognitive empathy, facial emotion recognition, and academic performance in adolescents enrolled in educational support programmes.

Facial emotion recognition involves multiple interacting perceptual, emotional, and socio-cognitive processes beyond overt behavioural accuracy. Previous electrophysiological studies have shown that neural responses to emotional faces emerge at early stages of processing and may index aspects of emotional processing that are not fully reflected in observable behavioural responses [80,81]. Neuroimaging research also suggests that emotional face processing may involve partially distinct neural and regulatory processes depending on the perceptual and interpretative demands of the task [82]. Accordingly, the exploratory electrophysiological differences observed in the present study should be considered complementary to, rather than confirmatory of, the behavioural findings. Furthermore, although cognitive empathy was significantly associated with FER performance, recent meta-analytic evidence indicates that the relationship between empathy and emotion recognition is generally modest and influenced by multiple moderating factors [47]. Facial emotion recognition and cognitive empathy may therefore represent related but distinct dimensions of socioemotional functioning, involving both perceptual and higher-order socio-cognitive processes [38,83].

### Differences in oscillatory activity between groups

Exploratory source-level estimates differed between groups across the theta, alpha, and beta frequency bands in broad occipital, temporal, parietal, and somatosensory cortical regions. The spatial distribution of the between-group differences varied across frequency bands and facial-expression conditions. Given the low-density EEG montage, these spatial patterns are described only in relation to broad, literature-based reference regions and do not establish focal cortical sources. Furthermore, because the overall group effect on facial emotion recognition accuracy was not statistically significant, the EEG differences should not be interpreted as neural evidence of impaired facial emotion recognition in the LAP group.

Previous studies have associated theta- and alpha-band activity with early facial processing, attentional regulation, and structural facial encoding [73,74,84,85], whereas beta-band activity has been linked to sensorimotor, temporoparietal, and higher-order socio-cognitive processes during emotion and social-perception tasks [75,86–90]. These processes are not specific to empathy. The observed between-group differences may also reflect variability in attentional control, emotional regulation, response selection, sensorimotor simulation, or other executive and socio-cognitive functions engaged during the task [74,82,87–91]. Given the interdependence of these functions during facial-expression processing, the present design cannot determine whether the observed differences are primarily related to empathy, emotion regulation, executive control, or broader task demands. Accordingly, these oscillatory patterns should not be considered specific markers of facial emotion recognition or empathy.

No associations between oscillatory activity and FER or empathy-related measures remained statistically significant after FDR correction. Consequently, the present data do not support conclusions about robust relationships between these electrophysiological, behavioural, and empathy-related measures.

Taken together, the EEG findings should be regarded as exploratory and complementary observations of task-related oscillatory activity. Replication in larger, independent samples and with higher-density EEG configurations is required before drawing stronger conclusions about the neurocognitive processes underlying the observed between-group differences.

## Conclusions

To our knowledge, no studies have compared the empathy levels of underachieving students in a reinforcement programme with those of a control group of students with adequate academic performance. As mentioned in the introduction, the groups in the learning and performance enhancement programme include students who cannot progress to the next educational level because of their grades.

Previous studies have characterised these students as having poor social skills, emotional instability, and maladaptive social relationships. In our study, students with low academic achievement who participated in learning enhancement programmes showed significantly lower scores in cognitive empathy, social values, justice and equality, personal values, and honesty. These results are consistent with previous studies on socioemotional functioning in LAP students. Furthermore, our study suggests that cognitive empathy, rather than affective empathy, may represent a particularly relevant socioemotional dimension in this group.

The overall group difference in facial emotion recognition accuracy was not statistically significant. Exploratory EEG analyses identified between-group differences in source-level oscillatory activity estimates that met the prespecified statistical threshold during facial-expression processing. These electrophysiological findings should not be interpreted as confirmation of a behavioural FER deficit, as precise anatomical localisations, or as specific neural correlates of reduced cognitive empathy. Overall, the self-report, behavioural, and electrophysiological measures provided complementary, rather than convergent, information about socioemotional and task-related functioning in this sample. Replication in larger, independent samples is required before drawing stronger neurocognitive or educational conclusions.

### Limitations

One limitation of this study concerns the sample size, although it falls within the range commonly reported in EEG studies on emotional processing, where samples typically include approximately 20–40 participants [42,56]. A sensitivity power analysis indicated that the available sample size was sufficient to detect large effects, but may have limited sensitivity for detecting smaller effects. It should be noted that the LAP group is relatively small and part of a specific educational support programme, which limited recruitment possibilities. The multimethod design provided complementary information from self-report, behavioural, and electrophysiological measures; however, it does not offset the limitations associated with the small sample, exploratory analyses, and cross-sectional design. In addition, although FER tasks offer controlled measures of emotion recognition, real-life social interactions involve more complex and dynamic cues; therefore, caution is warranted when generalising experimental findings to naturalistic social contexts.

An additional limitation concerns the interpretation of the electrophysiological findings. EEG-based measures of emotional face processing are sensitive to methodological decisions related to preprocessing, artefact correction, frequency-band selection, and analytical procedures. Differences in feature extraction strategies and signal-processing approaches may influence the oscillatory patterns identified and can limit comparability across studies. Importantly, the relatively low-density EEG montage used in this study (22 recording channels, plus reference and ground) limits the spatial resolution of source localisation. Therefore, the reported cortical regions should be interpreted as approximate areas compatible with those commonly associated with facial emotion processing, rather than precise or focal anatomical localisations. Recent reviews have highlighted the lack of fully standardised protocols in EEG research on emotion recognition, as well as challenges related to variability in experimental paradigms and analytical approaches [41,42].

Therefore, although the present study identified group differences in psychosocial and electrophysiological measures, the overall behavioural group effect on facial emotion recognition accuracy was not statistically significant. Although the EEG differences met the prespecified statistical threshold, their spatial and functional interpretation remains exploratory. They should therefore be understood as complementary indicators of task-related activity rather than as evidence of an empathy- or FER-specific neural mechanism. Moreover, emotion regulation and executive functioning were not directly assessed in the present study. Therefore, the extent to which the observed EEG differences reflect these broader processes, rather than empathy- or FER-specific mechanisms, cannot be determined. Future studies should directly assess emotion regulation and executive functioning using specific behavioural and neuropsychological measures. In addition, the EEG findings should be interpreted in light of methodological choices inherent to EEG analysis, including the use of power spectral density (PSD) as the main measure, the selected frequency bands, and the source localisation procedure. These analytical decisions may influence the spatial and functional interpretation of the observed oscillatory patterns. Although sex and age were controlled for in some analyses, the present study was not specifically designed to examine potential sex-related differences in empathy or facial emotion recognition. Future studies with larger samples should explore these variables in greater detail.

Because the participants were minors and the sample size was limited, other potentially relevant variables, such as socioeconomic status or cultural background, were not examined. Future studies with larger samples should consider these factors to better understand their relationship with empathy and academic achievement.

It is also important to note that empathy is a complex construct comprising multiple components and stages. The present study addressed some of these components at different levels; however, future research would benefit from more integrative approaches that capture the broader multidimensional nature of empathy.

Finally, future research would benefit from developing more ecologically valid paradigms that better capture the dynamic and context-dependent nature of emotional processes, as highlighted in recent discussions on the challenges of EEG-based emotion research [41]. Integrating neurophysiological, behavioural, and social-contextual measures may contribute to a more comprehensive understanding of empathy development in educational settings.

## Supporting information

S1 Text Supplementary EEG preprocessing quality-control procedures and results. (DOCX)

S1 Table Epoch rejection rates by group and emotional condition. (XLSX)

S2 Table Pre- to post-SSP mean absolute amplitude changes by EEG channel. (XLSX)

S3 Table Consolidated EEG preprocessing quality-control summaries of SSP-related amplitude changes, frontal EEG proxy indicators, residual post-SSP amplitude distributions, and quality-control information. (XLSX)

S1 Fig Representative blink-related EEG waveforms before and after SSP correction. (PDF)

S2 Text Sensitivity analyses of the focal psychosocial findings and the association between facial emotion recognition and cognitive empathy. (DOCX)

S3 Text Sensitivity analyses of source-level EEG group-contrast maps: candidate-outlier and participant-level leave-one-out analyses. (DOCX)

S2 Fig Candidate-outlier sensitivity of source-level EEG group-contrast maps across facial-expression conditions and theta, alpha, and beta frequency bands. (PDF)

S3 Fig Focused trial-level sensitivity analysis of source-level EEG group-contrast maps for Fear, Anger, and Disgust. (PDF)

S1 Data Individual-level psychometric data. (XLSX)

S2 Data Individual-level behavioural facial emotion recognition data. (XLSX)

S3 Data Individual-level EEG-derived PSD data and associated facial emotion recognition and empathy variables used in the EEG–behaviour correlation analyses. (XLSX)
