# Supplementary material for: Reduced cognitive empathy and differences in EEG activity in low-achieving adolescents
Source: PLoS One. 2026 Oct 27;21(10):e0361520. doi: 10.1371/journal.pone.0361520 (PMC13712686; doi:10.1371/journal.pone.0361520)
Supplement: Supplementary file 1 [file pone.0361520.s005.pdf]

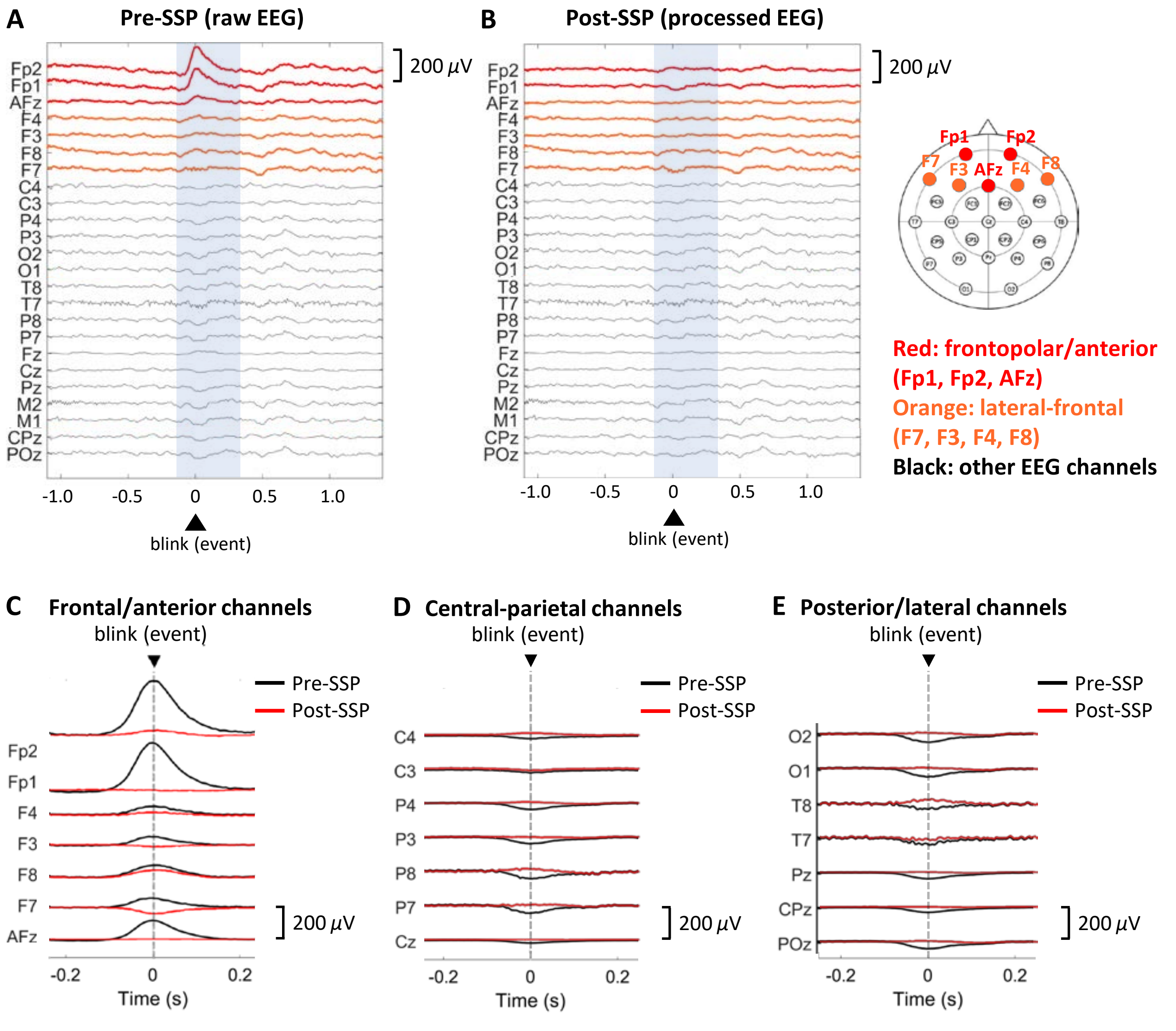

S1 Fig. Representative blink-related EEG waveforms before and after SSP correction. Panels A and B show the same representative blink-related EEG segment before and after SSP correction, using the same time window and amplitude scale. Panels C–E show blink-locked average waveforms for frontal/anterior, central–parietal, and posterior/lateral channel groups, respectively. Before SSP correction, blink-related activity was most prominent over anterior/frontal electrodes and was visibly attenuated after SSP correction, whereas posterior channels showed comparatively smaller changes.
