# Supplementary material for: Reduced cognitive empathy and differences in EEG activity in low-achieving adolescents
Source: PLoS One. 2026 Oct 27;21(10):e0361520. doi: 10.1371/journal.pone.0361520 (PMC13712686; doi:10.1371/journal.pone.0361520)
Supplement: Supplementary file 3 [file pone.0361520.s009.pdf]

S3 Fig. Focused trial-level sensitivity analysis of source-level EEG group-contrast maps for Fear, Anger, and Disgust.

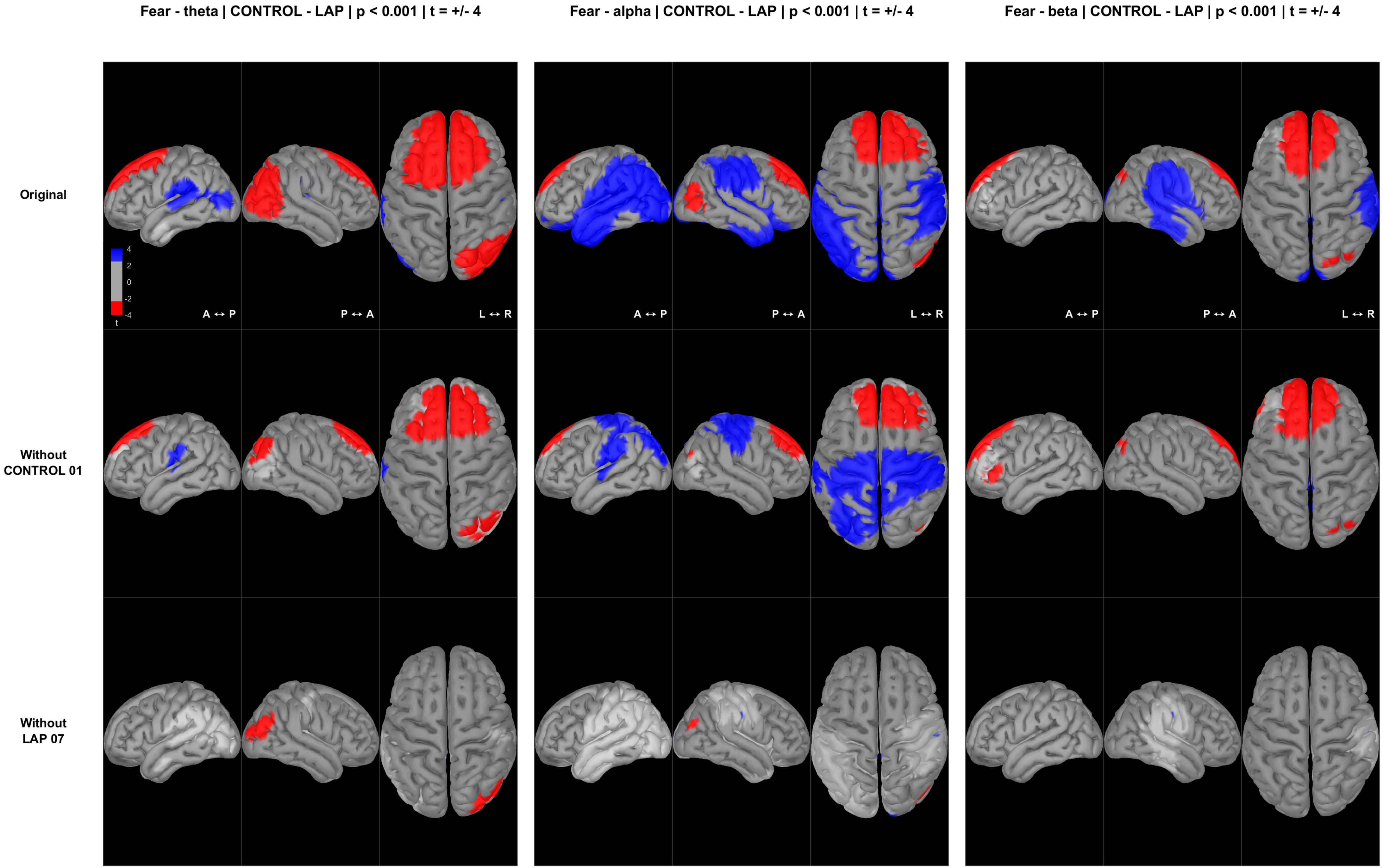

Red indicates higher source power in LAP than CONTROL, whereas blue indicates higher source power in CONTROL than LAP; maps are shown using a two-sided FDR-corrected threshold of $p < .001$ and a fixed t-value range from -4 to +4.

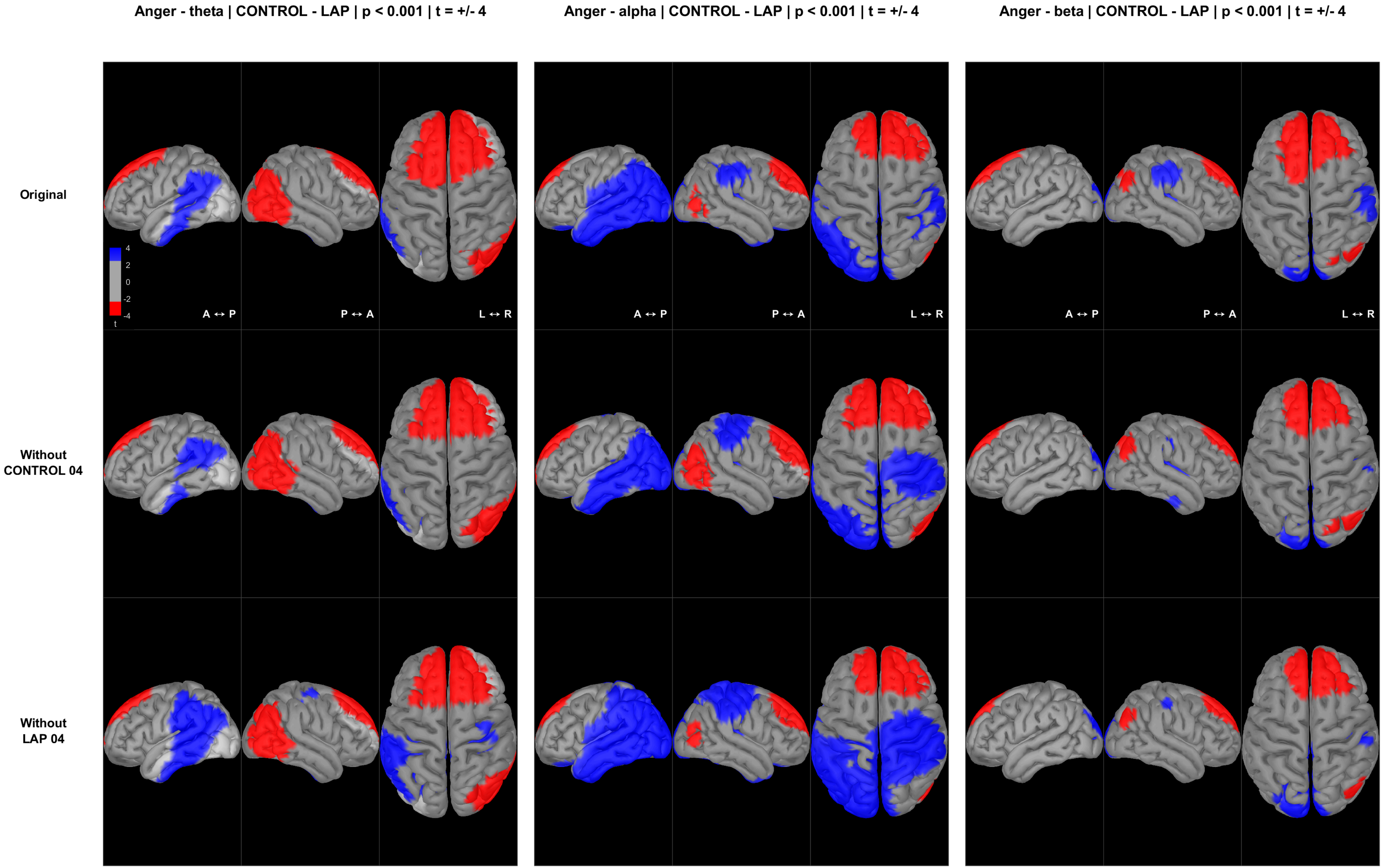

Red indicates higher source power in LAP than CONTROL, whereas blue indicates higher source power in CONTROL than LAP; maps are shown using a two-sided FDR-corrected threshold of p < .001 and a fixed t-value range from -4 to +4.

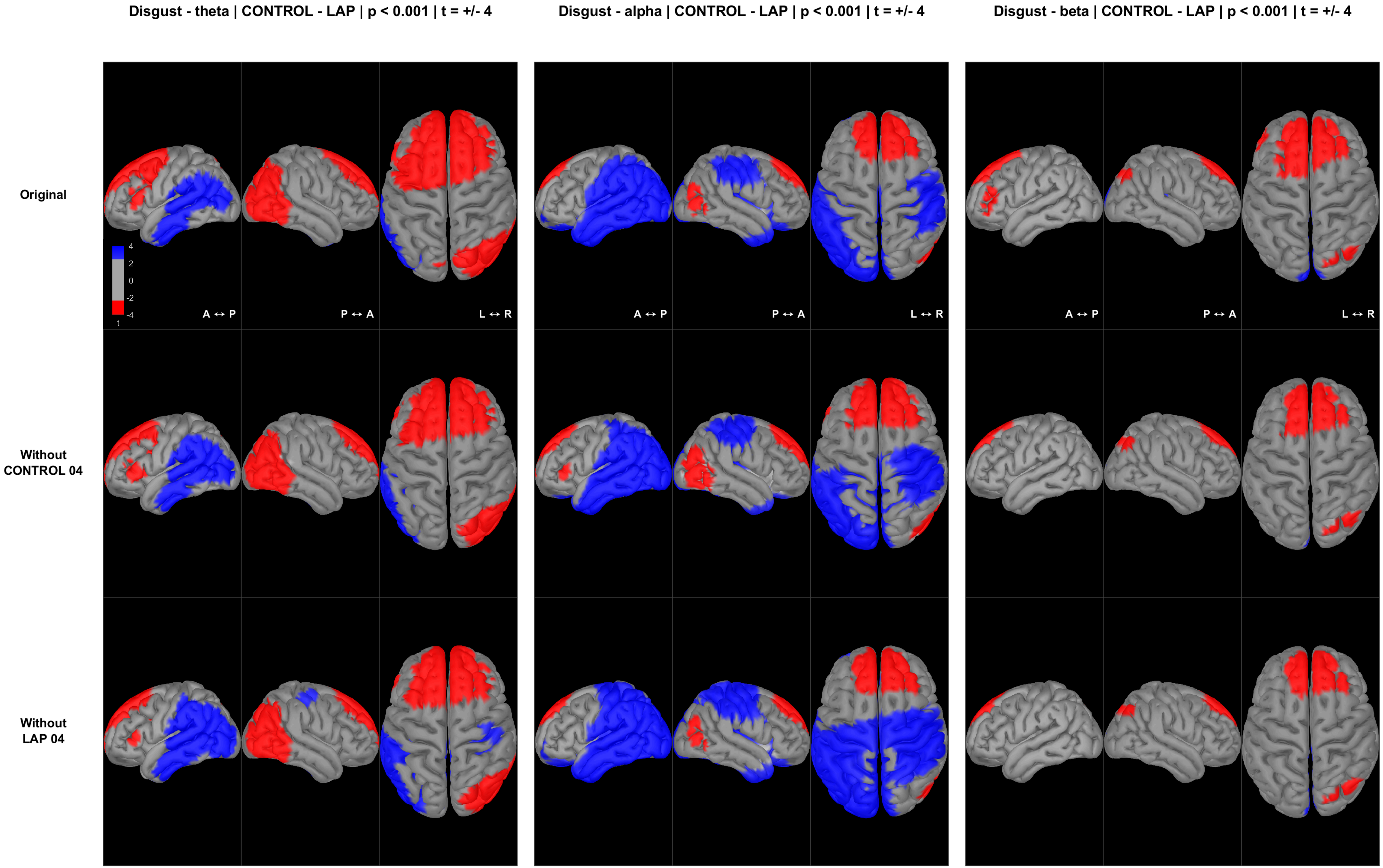

Red indicates higher source power in LAP than CONTROL, whereas blue indicates higher source power in CONTROL than LAP; maps are shown using a two-sided FDR-corrected threshold of $p < .001$ and a fixed t-value range from -4 to +4.
