# Supplementary material for: Reduced cognitive empathy and differences in EEG activity in low-achieving adolescents
Source: PLoS One. 2026 Oct 27;21(10):e0361520. doi: 10.1371/journal.pone.0361520 (PMC13712686; doi:10.1371/journal.pone.0361520)
Supplement: Supplementary file 4 [file pone.0361520.s001.docx]

S1 Text. Supplementary EEG preprocessing quality-control procedures and results

# Supplementary EEG preprocessing quality-control procedures

As an additional quality-control step for the EEG preprocessing pipeline, supplementary analyses were conducted to characterise epoch rejection rates, Signal-Space Projection (SSP)-related amplitude changes, frontal EEG proxy indicators of ocular-related activity, residual post-SSP amplitude distributions, and representative pre-/post-SSP waveforms. These analyses were implemented using custom MATLAB scripts applied to EEG data exported from Brainstorm.

# Data preparation and epoch matching

Equivalent pre-SSP and post-SSP epochs were exported for each participant, emotional condition, and trial using the same 0–2 s post-stimulus window used in the main EEG analyses. Pre- and post-SSP epochs were matched by group, participant, event, and trial before amplitude-based quality-control metrics were calculated. Analyses were restricted to the 24 EEG channels; gyroscope/movement channels and the status channel were excluded.

A total of 2,184 matched pre-/post-SSP epoch pairs were identified across the final EEG sample (control: n = 19; LAP: n = 20). To maintain consistency with the preprocessing decisions recorded in Brainstorm, the time interval of each exported epoch was reconstructed from the stimulus-event timing stored in the Brainstorm link-to-raw file. Epoch pairs were excluded when their 0–2 s interval overlapped an explicit BAD/QC interval in the same raw recording and could be matched unambiguously to the corresponding exported pre-/post-SSP pair. This temporal procedure identified 210 matched epoch pairs for exclusion, leaving 1,974 matched pairs for the amplitude-based analyses. At the channel level, channels marked as bad in either the pre-SSP or post-SSP file were excluded using Brainstorm ChannelFlag information; this removed 4,793 epoch–channel rows.

The epoch-rejection totals reported in S1 Table and the matched-epoch totals used for S2–S3 Tables serve related but distinct quality-control purposes. S1 Table summarises the complete epoch-rejection record according to the study preprocessing criteria, whereas S2–S3 Tables required exact temporal matching between the original raw BAD/QC intervals and available pre-/post-SSP epoch pairs. Consequently, the retained-epoch totals in these analyses are not expected to be identical.

# Epoch rejection rates

Epoch rejection rates were summarised by group and emotional condition (S1 Table). Each participant initially contributed eight epochs per emotional condition. Rejected and retained epochs were counted at participant and condition levels, and group summaries included the aggregated rejection rate and the mean participant-level rejection rate with its standard deviation.

# Amplitude-based quality-control metrics

Amplitude-based quality-control metrics were computed in MATLAB for each matched epoch and EEG channel after converting the Brainstorm-exported numerical signal values to microvolts. Mean absolute amplitude was calculated as the average of the absolute EEG signal values across the N time samples within an epoch:

*Mean absolute amplitude = (1/N) Σᵢ₌₁ᴺ |x(tᵢ)|*

where x(tᵢ) represents the EEG signal value at time sample i within the 0–2 s post-stimulus interval.

Peak-to-peak amplitude was calculated as the difference between the maximum and minimum signal values within the same epoch and channel:

*Peak-to-peak amplitude = max[x(t)] − min[x(t)]*

Pre- to post-SSP amplitude change was calculated separately for mean absolute amplitude and peak-to-peak amplitude:

*Pre–post SSP change = PreSSP − PostSSP*

Positive values therefore indicate lower amplitude after SSP, negative values indicate higher amplitude after SSP, and values close to zero indicate minimal pre- to post-SSP change. Between-group differences in SSP-related change were calculated as:

*LAP − control difference = ΔLAP − Δcontrol*

where Δ denotes the corresponding pre- to post-SSP change. Positive values indicate a larger SSP-related amplitude change in the LAP group, whereas negative values indicate a larger change in the control group. Metrics were first calculated at the epoch-by-channel level, then averaged within each participant, and finally summarised across participants so that each participant contributed equally to group estimates. Mean absolute amplitude results are reported in S2 Table and complementary peak-to-peak results in S3 Table.

# Frontal EEG proxy indicators of ocular-related activity

Because no dedicated electrooculography (EOG) channels were recorded, ocular-related activity was assessed indirectly using the spatial distribution of EEG amplitude changes across predefined channel sets (**S3 Table, Frontal_Proxy sheet).** The frontopolar core comprised Fp1 and Fp2; the orbito-frontal core comprised Fp1, Fp2, and AFz; and the broader frontal/anterior proxy comprised Fp1, Fp2, F3, F4, F7, F8, Fz, and AFz. These sets were compared with non-frontal, central–parietal, and posterior/lateral channel sets. Larger pre- to post-SSP changes over anterior channel sets than over non-frontal sets were interpreted as indirect spatial evidence compatible with attenuation of ocular-related components. These measures are EEG-based proxy indicators and should not be interpreted as dedicated EOG measurements or as direct estimates of blink frequency.

# Residual post-SSP amplitude distributions

Residual post-SSP amplitude distributions were examined to assess the signal remaining after SSP correction and to identify possible group imbalances or high-amplitude residual observations (S3 Table, Residual_Distribution sheet). Post-SSP mean absolute and peak-to-peak amplitudes were summarised by participant, group, and channel set. Distributional indices included the median, interquartile range, 95th and 99th percentiles, and maximum observed amplitude. Upper percentiles were emphasised because they describe the high-amplitude residual tail while being less dependent than the maximum on a single observation.

# Representative pre-/post-SSP waveforms

Representative blink-related EEG waveforms were displayed before and after SSP correction using identical time windows and amplitude scales (S1 Fig). Blink-locked averages were also shown for frontal/anterior, central–parietal, and posterior/lateral channel groups. The figure was intended as a qualitative illustration complementary to the quantitative amplitude summaries, rather than as an inferential group comparison.

# Supplementary quality-control results

Epoch rejection rates were closely comparable between groups (S1 Table). In the control group, 134 of 1,064 initial epochs were rejected, corresponding to an overall rejection rate of 12.59% (SD = 8.17), with 930 epochs retained. In the LAP group, 138 of 1,120 epochs were rejected, corresponding to an overall rejection rate of 12.32% (SD = 7.57), with 982 epochs retained. Across emotional conditions, rejection rates ranged from 9.21% to 15.13% in the control group and from 10.00% to 15.00% in the LAP group. The nearly identical overall rates and the overlapping condition-specific ranges indicate that the final EEG datasets were not substantially imbalanced by differential epoch rejection.

Mean absolute amplitude changes were spatially concentrated over anterior electrodes (S2 Table). The LAP-minus-control difference in pre- to post-SSP mean absolute amplitude change was 7.35 µV for the frontopolar core, 5.46 µV for the orbito-frontal core, and 2.80 µV for the broader frontal/anterior proxy. By contrast, the corresponding difference was −0.25 µV for the non-frontal scalp set and 0.82 µV across all scalp EEG channels excluding mastoids. The complementary peak-to-peak summaries showed the same general spatial pattern, with larger SSP-related changes over frontopolar and anterior channel sets and comparatively small between-group differences over non-frontal channels (S3 Table). Together with the frontal proxy summaries in S3 Table, this pattern suggests that SSP-related changes were predominantly anterior rather than uniformly distributed across the scalp. Given the absence of dedicated EOG channels, this finding is interpreted as indirect spatial evidence compatible with attenuation of blink-related or ocular-related activity, not as direct evidence of group differences in ocular artifacts.

Residual post-SSP amplitude distributions did not indicate a generalised between-group imbalance over the non-frontal scalp regions most relevant to the principal PSD findings (S3 Table). For mean absolute amplitude, the 95th percentile across all scalp channels excluding mastoids was 21.30 µV in the control group and 23.34 µV in the LAP group. In the non-frontal scalp set, the corresponding values were 22.33 and 22.46 µV, a LAP-minus-control difference of only 0.13 µV. Maximum values were considered descriptive because they may reflect single observations; interpretation therefore focused primarily on medians and upper percentiles. Consistent with these quantitative summaries, S1 Fig showed visible attenuation of blink-related activity over anterior/frontal electrodes after SSP correction, whereas central–parietal and posterior/lateral channels showed comparatively smaller changes.

Overall, the supplementary analyses document the effect of SSP-based artifact correction and support the broad comparability of the EEG data retained for the main analyses. Their purpose is methodological quality control: they should not be interpreted as independent evidence of neurophysiological group differences.
