# Supplementary material for: Reduced cognitive empathy and differences in EEG activity in low-achieving adolescents
Source: PLoS One. 2026 Oct 27;21(10):e0361520. doi: 10.1371/journal.pone.0361520 (PMC13712686; doi:10.1371/journal.pone.0361520)
Supplement: Supplementary file 5 [file pone.0361520.s006.docx]

S2 Text. Sensitivity analyses of the focal psychosocial findings and the association between facial emotion recognition and cognitive empathy

# Overview

These supplementary analyses evaluated the robustness of the five focal psychosocial between-group findings and the association between facial emotion recognition (FER) and cognitive empathy. The analyses comprised potential-outlier sensitivity checks, leave-one-out analyses, and adjustment of the focal psychosocial outcomes for age and sex. All observations were retained in the primary analyses; exclusions were made only within the corresponding sensitivity analyses.

# Supplementary sensitivity-analysis procedures

## Potential-outlier sensitivity of focal psychosocial outcomes

Potential outliers were identified separately within the CONTROL and LAP groups for each focal outcome using Tukey’s rule. Observations below Q1 - 1.5×IQR or above Q3 + 1.5×IQR were flagged. For each sensitivity analysis, only the observations flagged for the outcome under consideration were excluded, and the Mann-Whitney U comparison was repeated. False discovery rate (FDR) adjustment was recalculated across the complete family of 18 psychosocial outcomes using the Benjamini-Hochberg procedure. For justice and equality, the exact recalculated value was p = .0000506; however, p = .001 was entered into the FDR procedure as a conservative representation of the original SigmaPlot output, which reported p < .001. Rank-biserial r was used as the effect-size estimate, with positive values indicating higher scores in CONTROL than in LAP.

## Leave-one-out sensitivity of focal psychosocial outcomes

Each participant was omitted in turn, producing 40 leave-one-out iterations for each focal outcome. The Mann-Whitney U comparison and the Benjamini-Hochberg FDR adjustment across the 18-outcome family were recalculated in every iteration. Stability was summarised using the range of FDR-adjusted p values, the range of rank-biserial correlations, the number of iterations retaining FDR-adjusted significance, and the number of effect-direction reversals.

## Adjustment for age and sex

For each focal psychosocial outcome, a separate linear regression model was fitted with group, age, and sex as predictors. Negative group coefficients indicate lower adjusted scores in LAP. Heteroskedasticity-consistent HC3 standard errors, 95% confidence intervals, and two-sided robust Wald tests were used. FDR adjustment was applied across the five adjusted group coefficients. All 40 participants had complete data for these analyses.

## Sensitivity of the FER-cognitive empathy association

Potential outliers were identified separately within group for FER performance and cognitive empathy using the same 1.5×IQR rule. The focal association was estimated using a partial Spearman correlation controlling for age, sex, and group. FER, cognitive empathy, and the covariates were rank-transformed, and the residuals of ranked FER and ranked cognitive empathy were correlated after adjustment for the ranked covariates. FDR adjustment was recalculated across the family of three FER-empathy correlations (affective, cognitive, and total). A complementary leave-one-out analysis then omitted each participant in turn and recalculated the adjusted correlation and the three-correlation FDR family in each of 40 iterations.

# Supplementary results

## Potential-outlier sensitivity of focal psychosocial outcomes

All five focal between-group differences retained their original direction and remained statistically significant after FDR adjustment when observations flagged by the prespecified rule were excluded. Cognitive empathy showed a slightly larger rank-biserial effect after exclusion. The social-values effect was attenuated but remained significant. No potential outliers were identified for justice and equality, personal values, or honesty.

Figure A. Distribution of the five focal psychosocial outcomes by group and observations flagged by the within-group 1.5×IQR rule. Individual observations are shown with boxplots; red circles indicate observations flagged as potential outliers. All observations were retained in the primary analyses.

Table A. Between-group results for the five focal psychosocial outcomes with and without potential outliers.

| **Outcome** | **Analysis** | **N CONTROL/LAP** | **CONTROL median [Q1-Q3]** | **LAP median [Q1-Q3]** | **U** | **p** | **FDR-adjusted p** | **Rank-biserial r** | **Excluded observations** |
| --- | --- | --- | --- | --- | --- | --- | --- | --- | --- |
| Cognitive empathy | Full sample | 20/20 | 21.00 [20.00-22.50] | 18.00 [15.00-19.25] | 90.00 | .0028 | .026 | .550 | - |
| Cognitive empathy | Sensitivity | 19/18 | 21.00 [20.00-23.00] | 18.00 [17.00-19.75] | 76.00 | .0037 | .033 | .556 | CONTROL-09; LAP-03; LAP-08 |
| Social values | Full sample | 20/20 | 45.00 [43.00-50.00] | 42.00 [40.00-43.50] | 89.00 | .0077 | .028 | .507 | - |
| Social values | Sensitivity | 20/19 | 45.00 [43.00-50.00] | 42.00 [40.00-43.75] | 89.00 | .013 | .047 | .480 | LAP-03 |
| Justice and equality | Full sample | 20/20 | 20.00 [17.00-20.00] | 15.00 [13.50-17.00] | 43.00 | .000051 | .018 | .762 | - |
| Justice and equality | Sensitivity | 20/20 | 20.00 [17.00-20.00] | 15.00 [13.50-17.00] | 43.00 | .000051 | .018 | .762 | None |
| Personal values | Full sample | 20/20 | 50.00 [48.00-56.50] | 47.00 [41.00-53.00] | 87.50 | .0067 | .028 | .515 | - |
| Personal values | Sensitivity | 20/20 | 50.00 [48.00-56.50] | 47.00 [41.00-53.00] | 87.50 | .0067 | .033 | .515 | None |
| Honesty | Full sample | 20/20 | 19.00 [18.00-20.00] | 17.00 [14.50-18.00] | 89.50 | .0073 | .028 | .504 | - |
| Honesty | Sensitivity | 20/20 | 19.00 [18.00-20.00] | 17.00 [14.50-18.00] | 89.50 | .0073 | .033 | .504 | None |

**Note.** Summary values are medians [Q1-Q3]. Potential outliers were removed only in the sensitivity rows. For justice and equality, p = .001 rather than the exact recalculated p value was used in the FDR procedure.

## Leave-one-out sensitivity of focal psychosocial outcomes

The five focal outcomes remained significant after FDR adjustment in all 40 leave-one-out iterations. No effect-direction reversals were observed. The maximum FDR-adjusted p value was below .05 for every outcome, indicating that the rank-based focal comparisons were not dependent on any single participant.

Table B. Leave-one-out sensitivity of the five focal psychosocial outcomes.

| **Outcome** | **Iterations** | **FDR-significant iterations** | **FDR-adjusted p range** | **Rank-biserial r range** | **Sign reversals** | **Conclusion** |
| --- | --- | --- | --- | --- | --- | --- |
| Cognitive empathy | 40 | 40 (100%) | .0068-.0435 | .526-.629 | 0 | Stable in every iteration |
| Social values | 40 | 40 (100%) | .0173-.0467 | .480-.573 | 0 | Stable in every iteration |
| Justice and equality | 40 | 40 (100%) | .0003-.0016 | .749-.819 | 0 | Stable in every iteration |
| Personal values | 40 | 40 (100%) | .0168-.0467 | .488-.585 | 0 | Stable in every iteration |
| Honesty | 40 | 40 (100%) | .0151-.0467 | .477-.576 | 0 | Stable in every iteration |

**Note.** Mann-Whitney U tests were repeated after omitting each participant in turn. FDR adjustment was recalculated across the full family of 18 psychosocial comparisons in every iteration.

## Adjustment for age and sex

The groups differed substantially in age, whereas the male/female distribution did not differ significantly. After adjustment for age and sex, justice and equality, honesty, social values, and personal values retained negative LAP-minus-CONTROL coefficients and remained significant after FDR adjustment. The adjusted cognitive-empathy coefficient was attenuated and its confidence interval included zero. The absolute magnitude of the cognitive-empathy group coefficient decreased by 47.3% after adjustment for age and sex.

Table C, Panel A. Demographic characteristics relevant to the sensitivity analysis.

| **Characteristic** | **CONTROL** | **LAP** | **Comparison** | **p** |
| --- | --- | --- | --- | --- |
| N | 20 | 20 | - | - |
| Age, mean (SD), years | 14.81 (0.46) | 16.09 (1.06) | Welch t-test | < .001 |
| Sex, male/female, n | 8/12 | 11/9 | Fisher exact test | .527 |

**Note.** The age comparison describes the covariate imbalance and was not one of the five focal outcome tests.

Table C, Panel B. Age- and sex-adjusted regression models for the five focal psychosocial outcomes.

| **Outcome** | **N** | **Adjusted group b (LAP-CONTROL)** | **HC3 SE** | **Wald z** | **p** | **FDR-adjusted p** | **95% CI for b** | **R²** | **Interpretation** |
| --- | --- | --- | --- | --- | --- | --- | --- | --- | --- |
| Cognitive empathy | 40 | -2.004 | 1.890 | -1.060 | .289 | .289 | [-5.707, 1.700] | .264 | Not retained |
| Justice and equality | 40 | -5.214 | 1.270 | -4.105 | < .001 | < .001 | [-7.704, -2.725] | .473 | Retained after FDR |
| Honesty | 40 | -3.012 | 1.262 | -2.387 | .017 | .028 | [-5.485, -0.539] | .236 | Retained after FDR |
| Social values | 40 | -8.608 | 3.898 | -2.208 | .027 | .034 | [-16.248, -0.967] | .267 | Retained after FDR |
| Personal values | 40 | -8.800 | 3.428 | -2.567 | .010 | .026 | [-15.519, -2.080] | .269 | Retained after FDR |

**Note.** Each row represents a separate model: outcome = group + age + sex. Negative coefficients indicate lower adjusted scores in LAP. R² refers to the complete model.

Table C, Panel C. Focused assessment of age and the cognitive-empathy group difference.

| **Analysis** | **Estimate** | **HC3 SE** | **95% CI** | **p** | **Model R²** | **Interpretation** |
| --- | --- | --- | --- | --- | --- | --- |
| Age vs cognitive empathy, complete sample | Spearman ρ = -.443 | - | - | .004 | - | Negative zero-order association |
| Unadjusted group coefficient | b = -3.800 | 1.231 | [-6.213, -1.387] | .002 | .209 | Lower cognitive empathy in LAP |
| Group coefficient adjusted for age | b = -2.018 | 1.845 | [-5.633, 1.598] | .274 | .263 | 46.9% attenuation; not significant |
| Group coefficient adjusted for age and sex | b = -2.004 | 1.890 | [-5.707, 1.700] | .289 | .264 | 47.3% attenuation; not significant |
| Age coefficient in the full model | b = -1.144 | 0.896 | [-2.900, 0.611] | .201 | .264 | Age not independently significant |

**Note.** Within-group Spearman correlations between age and cognitive empathy were not significant (CONTROL: ρ = .297, p = .204; LAP: ρ = -.255, p = .277). The attenuation is consistent with demographic overlap or confounding, but the sample does not establish age as an independent causal explanation.

## Sensitivity of the FER-cognitive empathy association

In the complete sample, FER performance was positively associated with cognitive empathy after adjustment for age, sex, and group (partial Spearman ρ = .411, p = .0114, FDR-adjusted p = .034). After excluding the five observations flagged for FER or cognitive empathy, the association retained its positive direction but was attenuated and no longer statistically significant (ρ = .331, p = .0558, FDR-adjusted p = .167). The focal association was therefore sensitive to the potential-outlier rule.

Table D. Partial Spearman association between FER performance and cognitive empathy with and without potential outliers.

| **Analysis** | **N** | **Partial Spearman ρ** | **t** | **df** | **p** | **FDR-adjusted p** | **Excluded observations** | **Interpretation** |
| --- | --- | --- | --- | --- | --- | --- | --- | --- |
| Full sample | 40 | .411 | 2.671 | 35 | .0114 | .034 | - | Significant after FDR |
| Sensitivity | 35 | .331 | 1.985 | 30 | .0558 | .167 | CONTROL-O1; CONTROL-02; LAP-O1; LAP-O2; LAP-03 | Direction retained; not significant |

**Note.** Partial Spearman correlations controlled for age, sex, and group. FDR adjustment was calculated across the three FER-empathy correlations. All observations were retained in the primary analysis.

Figure B. Sensitivity of the FER-cognitive empathy association to observations flagged by the within-group 1.5×IQR rule. Panels show the distributions of cognitive empathy and FER, the adjusted association in the complete sample, and the association after exclusion of the five flagged observations.

## Leave-one-out sensitivity of the FER-cognitive empathy association

The partial correlation remained positive in all 40 leave-one-out iterations, with no sign reversals. FDR-adjusted significance was retained in 31 iterations (77.5%). The correlation ranged from .350 to .488, whereas the FDR-adjusted p value ranged from .0077 to .1100. Thus, the direction and moderate magnitude of the association were stable, but its FDR-adjusted statistical significance showed moderate sensitivity to individual omissions.

Table E. Leave-one-out sensitivity of the FER-cognitive empathy association.

| **Association** | **Iterations** | **FDR-significant iterations** | **ρ range** | **FDR-adjusted p range** | **Sign reversals** | **Interpretation** |
| --- | --- | --- | --- | --- | --- | --- |
| FER-cognitive empathy | 40 | 31 (77.5%) | .350-.488 | .0077-.1100 | 0 | Positive direction in all iterations; FDR-adjusted significance moderately sensitive to individual omissions. |

**Note.** Partial Spearman correlations controlled for age, sex, and group. FDR adjustment was recomputed across the three FER-empathy correlations in each iteration.

# Overall interpretation

The five focal psychosocial between-group differences were robust to the potential-outlier and leave-one-out procedures. Four outcomes - justice and equality, honesty, social values, and personal values - also remained significant after adjustment for age and sex. Cognitive empathy was the exception: its unadjusted between-group difference was robust to individual exclusions but was substantially attenuated after demographic adjustment. The FER-cognitive empathy association retained a positive direction across the potential-outlier and leave-one-out analyses, although its statistical significance was sensitive to both the prespecified outlier rule and some individual omissions. These sensitivity findings support cautious interpretation of cognitive empathy and the FER-cognitive empathy association while strengthening the robustness of the four values-related group differences.
