# Supplementary material for: Reduced cognitive empathy and differences in EEG activity in low-achieving adolescents
Source: PLoS One. 2026 Oct 27;21(10):e0361520. doi: 10.1371/journal.pone.0361520 (PMC13712686; doi:10.1371/journal.pone.0361520)
Supplement: Supplementary file 6 [file pone.0361520.s007.docx]

S3 Text. Candidate-outlier and participant-level leave-one-out sensitivity analyses of the EEG group-contrast maps

# Overview

Two complementary sensitivity frameworks were used to examine whether the exploratory CONTROL–LAP EEG group-contrast patterns were disproportionately influenced by a small number of participants. The first retained the original trial-level statistical framework and recalculated thresholded source-level maps after removal of prespecified candidate outliers. The second was a participant-level leave-one-out analysis of unthresholded spatial t-map patterns. These analyses address related but distinct questions and should not be interpreted as interchangeable replications of the same statistical model. Table A presents the candidate-outlier analyses, whereas Table B presents the participant-level leave-one-out analyses.

# Supplementary sensitivity-analysis procedures

## Candidate-outlier sensitivity within the original trial-level framework

Candidate participants were identified from participant-level EEG summaries using global, spatial, and combined criteria. Five participants were examined: CONTROL-04, LAP-17, CONTROL-01, CONTROL-21, and LAP-11. Candidate status triggered sensitivity analysis only; none of these participants was automatically removed from the primary analysis. The original trial-level CONTROL–LAP analysis was repeated after excluding each candidate separately and after excluding all five simultaneously. The candidate-identification criteria and participant-level summary values are reported in Table A, Panel A. The “Global criterion,” “Spatial criterion,” and “Combined criterion” columns show why each participant was selected, while “Combined RMS score” and “Extreme emotion × band cells” describe the magnitude and extent of the atypical EEG summaries. The robustness outputs from the individual and combined exclusions are reported in Table A, Panels B and C.

For the spatial displays and quantitative summaries, theta-, alpha-, and beta-band maps were shown using a two-sided FDR-corrected threshold of p < .001 and a fixed t-value range from −4 to +4. Red indicates higher source power in LAP than CONTROL, whereas blue indicates higher source power in CONTROL than LAP. Map correlation quantified similarity between the complete-sample and sensitivity t-value maps; sign agreement quantified preservation of the CONTROL–LAP direction; the significant-vertex ratio quantified change in suprathreshold spatial extent; and the Jaccard index quantified overlap between suprathreshold vertices. Table A, Panel B summarises these metrics in the “Median map correlation,” “Minimum map correlation,” “Median sign agreement (%),” “Median significant-vertex ratio,” and “Median Jaccard index” columns. Table A, Panel C reports the condition-by-band values in the “All-five map r,” “Sign agreement (%),” “Significant-vertex ratio,” and “Jaccard” columns; the “Worst single exclusion” and “Minimum single r” columns identify the candidate producing the largest map change for each condition and band.

## Participant-level leave-one-out analysis of unthresholded maps

Each of the 39 participants in the final EEG sample was omitted in turn. For every omission, CONTROL–LAP participant-level t-maps were recalculated for seven facial-expression conditions and the theta, alpha, and beta bands, yielding 819 leave-one-out maps. Each map was compared with the corresponding complete-sample participant-level map using spatial correlation and sign agreement. This analysis assessed the stability of the unthresholded spatial pattern and whether any single participant dominated the group contrast. The aggregate warning frequencies are reported in the “Result” column of Table B, Panel A. The “Map correlation,” “Sign agreement,” and “Interpretation” columns of Table B, Panel B describe the magnitude and nature of the most influential participant-by-condition combinations.

None of the 21 complete-sample participant-level theta-, alpha-, or beta-band maps contained vertices at p < .001. Consequently, Jaccard indices and significant-vertex ratios were of limited interpretive value in this framework: when both maps contain no suprathreshold vertices, a Jaccard value of 1 does not establish that the underlying unthresholded topographies are identical. Spatial correlation and sign agreement were therefore treated as the principal robustness measures. The meaning and analytical role of each robustness metric are summarised in the “What it measures” and “Use in this analysis” columns of Table B, Panel C.

## Focused trial-level visual sensitivity analysis

A focused trial-level analysis was additionally used to visualise selected influential and stable participant-condition combinations. For Fear, maps were recalculated after omission of CONTROL-01 and LAP-07. Anger and Disgust were included as comparatively stable reference conditions and were recalculated after omission of CONTROL-04 and LAP-04. This focused thresholded analysis is distinct from the 819-map participant-level leave-one-out summary and is presented as a targeted spatial illustration rather than as its direct visual equivalent.

# Supplementary results

## Candidate-outlier sensitivity results

Across all available emotion-by-frequency maps, removal of individual candidate outliers generally preserved the spatial pattern of the complete-sample contrast. Median map correlations ranged from .988 to .998 for the individual exclusions. Simultaneous removal of all five candidates produced a lower but still high median map correlation of .949 and median sign agreement of 90.3%. The principal exception was the Fear condition, for which theta-, alpha-, and beta-band maps showed marked sensitivity to exclusion of the candidate participants, especially CONTROL-01. Several beta-band maps also showed reduced sign agreement or reduced overlap in suprathreshold extent despite high map correlations. The overall exclusion results are summarised in the “Median map correlation,” “Minimum map correlation,” and “Median sign agreement (%)” columns of Table A, Panel B. The emotion-by-band pattern underlying the Fear and beta-band qualifications is detailed in the “All-five map r,” “Sign agreement (%),” “Significant-vertex ratio,” and “Jaccard” columns of Table A, Panel C. The “Worst single exclusion” and “Minimum single r” columns identify CONTROL-01 as the most influential candidate for Fear across all three bands.

**Main candidate-outlier finding: the overall trial-level EEG topography was broadly stable, but the Fear condition showed clear sensitivity and selected beta-band maps showed sensitivity in suprathreshold extent.**

Table A, Panel A. Candidate outliers identified in participant-level EEG summaries.

| **Participant** | **Group** | **Global criterion** | **Spatial criterion** | **Combined criterion** | **Combined RMS score** | **Extreme emotion × band cells** |
| --- | --- | --- | --- | --- | --- | --- |
| CONTROL-04 | CONTROL | Yes | Yes | Yes | 3.2945 | 32 |
| LAP-17 | LAP | No | Yes | Yes | 3.2689 | 24 |
| CONTROL-01 | CONTROL | Yes | No | Yes | 3.1910 | 13 |
| CONTROL-21 | CONTROL | No | Yes | Yes | 2.9551 | 14 |
| LAP-11 | LAP | No | Yes | No | 1.7225 | 7 |

**Note.** Candidate status was based on participant-level global, spatial, and combined EEG summary criteria. Candidates were examined in sensitivity analyses and were retained in the primary analysis.

Table A, Panel B. Overall map robustness after removal of candidate outliers.

| **Exclusion scenario** | **Median map correlation** | **Minimum map correlation** | **Median sign agreement (%)** | **Median significant-vertex ratio** | **Median Jaccard index** |
| --- | --- | --- | --- | --- | --- |
| Without LAP11 | .99811 | .91963 | 98.674 | .97902 | .89186 |
| Without CONTROL-21 | .99310 | .93675 | 98.707 | .93613 | .83496 |
| Without LAP17 | .99051 | .76968 | 98.637 | 1.08810 | .82238 |
| Without CONTROL-04 | .98953 | .81446 | 97.220 | .88867 | .76805 |
| Without CONTROL-01 | .98792 | −.06764 | 98.100 | 1.00240 | .78482 |
| Without all five | .94940 | −.04594 | 90.295 | .89460 | .54331 |

**Note.** Values summarise all emotion × frequency-band maps in the full candidate-outlier sensitivity output. Panel C focuses on theta, alpha, and beta, matching S2 Fig.

Table A, Panel C. Robustness summary by facial-expression condition and frequency band after simultaneous removal of all five candidate outliers.

| **Condition** | **Band** | **Original significant vertices** | **All-five map r** | **Sign agreement (%)** | **Significant-vertex ratio** | **Jaccard** | **Worst single exclusion** | **Minimum single r** |
| --- | --- | --- | --- | --- | --- | --- | --- | --- |
| Happiness | Theta | 7217 | 0.951 | 88.6 | 0.960 | 0.722 | Without CONTROL-21 | 0.967 |
| Happiness | Alpha | 11063 | 0.944 | 85.6 | 0.777 | 0.631 | Without CONTROL-04 | 0.956 |
| Happiness | Beta | 7674 | 0.971 | 76.3 | 0.568 | 0.493 | Without LAP17 | 0.969 |
| Neutral | Theta | 9446 | 0.949 | 88.1 | 0.869 | 0.692 | Without CONTROL-21 | 0.972 |
| Neutral | Alpha | 11868 | 0.959 | 88.4 | 0.893 | 0.753 | Without CONTROL-04 | 0.960 |
| Neutral | Beta | 5461 | 0.912 | 76.8 | 0.506 | 0.405 | Without LAP17 | 0.932 |
| Sadness | Theta | 10323 | 0.970 | 90.0 | 0.813 | 0.670 | Without CONTROL-21 | 0.978 |
| Sadness | Alpha | 11809 | 0.972 | 88.8 | 0.881 | 0.729 | Without CONTROL-04 | 0.974 |
| Sadness | Beta | 5389 | 0.940 | 76.5 | 1.012 | 0.674 | Without LAP17 | 0.957 |
| Surprise | Theta | 10374 | 0.969 | 92.5 | 0.896 | 0.701 | Without LAP17 | 0.977 |
| Surprise | Alpha | 12331 | 0.952 | 90.6 | 0.927 | 0.803 | Without CONTROL-04 | 0.957 |
| Surprise | Beta | 8933 | 0.968 | 87.3 | 0.642 | 0.495 | Without LAP17 | 0.982 |
| Fear | Theta | 1649 | 0.127 | 28.0 | 1.658 | 0.098 | Without CONTROL-01 | 0.278 |
| Fear | Alpha | 4316 | 0.354 | 34.9 | 1.081 | 0.530 | Without CONTROL-01 | 0.411 |
| Fear | Beta | 4317 | 0.254 | 42.2 | 0.943 | 0.038 | Without CONTROL-01 | 0.278 |
| Anger | Theta | 8192 | 0.956 | 93.1 | 0.819 | 0.709 | Without CONTROL-21 | 0.978 |
| Anger | Alpha | 11345 | 0.954 | 86.0 | 0.866 | 0.711 | Without CONTROL-04 | 0.956 |
| Anger | Beta | 6532 | 0.932 | 80.7 | 0.619 | 0.357 | Without LAP17 | 0.963 |
| Disgust | Theta | 8557 | 0.958 | 93.4 | 0.934 | 0.724 | Without CONTROL-21 | 0.969 |
| Disgust | Alpha | 11956 | 0.951 | 91.6 | 0.897 | 0.806 | Without CONTROL-04 | 0.954 |
| Disgust | Beta | 5687 | 0.824 | 83.8 | 0.709 | 0.363 | Without LAP17 | 0.810 |

**Note.** Map correlations compare sensitivity maps with the corresponding complete-sample trial-level map. “Worst single exclusion” identifies the individual candidate exclusion with the lowest map correlation for that condition and band. Statistical display: FDR-corrected threshold p < .001; fixed t range −4 to +4. High overall robustness was observed for Happiness theta; Neutral alpha; Sadness alpha; Surprise theta and alpha; Anger theta and alpha; and Disgust theta and alpha. Marked sensitivity was concentrated in Fear and selected beta-band maps.

## Participant-level leave-one-out results

The participant-level leave-one-out maps were highly stable overall. Only 6 of 819 maps had a spatial correlation below .90, 5 had a correlation below .80, and 3 had sign agreement below 80%. Because the three low-sign-agreement maps were among the six maps with correlation below .90, 813 of 819 maps (99.27%) showed neither warning signal. The clearest sensitivity occurred for Fear after removal of CONTROL-01. Omission of LAP-07 produced a secondary topographic redistribution in Fear theta and alpha, while LAP-17 produced a smaller secondary sensitivity in Disgust beta. The aggregate warning counts are reported in the “Result” column of Table B, Panel A. The “Map correlation” and “Sign agreement” columns of Table B, Panel B quantify the departures associated with each listed case, while “Interpretation” distinguishes directional change from topographic redistribution.

**Main leave-one-out finding: 813 of 819 unthresholded participant-level maps (99.27%) showed neither spatial correlation below .90 nor sign agreement below 80%.**

Table B, Panel A. Overall stability across the 39 participant omissions.

| **Indicator** | **Result** | **Interpretation** |
| --- | --- | --- |
| Participants | 39 | Final EEG sample |
| Leave-one-out maps evaluated | 819 | 39 participants × 7 conditions × 3 bands |
| Spatial correlation < .90 | 6/819 (0.73%) | Rare departures from the complete-sample topography |
| Spatial correlation < .80 | 5/819 (0.61%) | Strong changes localised to a small number of cases |
| Sign agreement < 80% | 3/819 (0.37%) | Direction usually preserved |
| Maps without either warning signal | 813/819 (99.27%) | Very high overall robustness |

**Note.** The value 813/819 is the corrected count corresponding to 99.27%. The previously displayed value 792/819 was a transcription error.

Table B, Panel B. Most influential participant-by-condition combinations in the participant-level leave-one-out analysis.

| **Rank** | **Participant** | **Group** | **Condition** | **Band** | **Map correlation** | **Sign agreement** | **Interpretation** |
| --- | --- | --- | --- | --- | --- | --- | --- |
| 1 | CONTROL-01 | CONTROL | Fear | Beta | .209 | 48.5% | Strong topographic and directional change |
| 2 | CONTROL-01 | CONTROL | Fear | Theta | .318 | 36.2% | Strong topographic and directional change |
| 3 | CONTROL-01 | CONTROL | Fear | Alpha | .444 | 43.5% | Strong topographic and directional change |
| 4 | LAP-07 | LAP | Fear | Alpha | .489 | 96.1% | Topographic redistribution with preserved direction |
| 5 | LAP-07 | LAP | Fear | Theta | .512 | 86.4% | Moderate topographic sensitivity |
| 6 | LAP-17 | LAP | Disgust | Beta | .827 | 88.8% | Secondary sensitivity |

Table B, Panel C. Definition and interpretation of participant-level leave-one-out robustness metrics.

| **Metric** | **What it measures** | **Use in this analysis** |
| --- | --- | --- |
| Spatial correlation | Similarity of the complete vertex-wise t-map before and after an omission | Primary indicator of topographic stability |
| Sign agreement | Percentage of vertices retaining the same CONTROL–LAP direction | Distinguishes redistribution from directional reversal |
| Jaccard index | Overlap between suprathreshold vertices in the two maps | Limited here because complete-sample participant-level maps had no vertices at p < .001 |
| Significant-vertex ratio | Change in the number of suprathreshold vertices | Useful only when an omission creates or removes significant vertices |

**Note.** The participant-level analysis assessed unthresholded t-map stability. It should not be interpreted as a test of persistence of suprathreshold clusters.

## Interpretation across the two sensitivity frameworks

The two frameworks converged in indicating that most EEG spatial patterns were not dominated by a single participant. However, they also identified a localised vulnerability of the Fear condition. In the original trial-level framework, simultaneous removal of the five candidate outliers substantially altered the Fear maps across theta, alpha, and beta. In the participant-level leave-one-out framework, the same condition accounted for five of the six maps with spatial correlation below .90, with CONTROL-01 producing the strongest topographic and directional changes. The trial-level Fear sensitivity is quantified in the Fear rows of Table A, Panel C, particularly in the “All-five map r,” “Sign agreement (%),” and “Jaccard” columns. The corresponding participant-level warnings are summarised in the “Result” column of Table B, Panel A, and the influential cases are detailed through the “Map correlation,” “Sign agreement,” and “Interpretation” columns of Table B, Panel B.

These findings support describing the overall EEG pattern as broadly robust while explicitly identifying the Fear results as sensitive to a small number of influential observations. They do not justify removing these participants retrospectively from the primary analysis, nor do they establish that the remaining spatial patterns represent precise anatomical or function-specific effects.

# Associated supplementary figures

S2 Fig. Candidate-outlier sensitivity of source-level EEG group-contrast maps across facial-expression conditions and theta, alpha, and beta frequency bands. Rows show the complete-sample map, simultaneous removal of all five candidate outliers, and removal of each candidate separately. Red indicates higher source power in LAP than CONTROL, whereas blue indicates higher source power in CONTROL than LAP. Maps are displayed at FDR-corrected threshold p < .001 with a fixed t range from −4 to +4.

S3 Fig. Focused trial-level EEG sensitivity analysis for selected participant-condition combinations in Fear, Anger, and Disgust. Fear maps are shown for the complete sample and after omission of CONTROL-01 and LAP-07. Anger and Disgust provide comparatively stable reference conditions after omission of CONTROL-04 and LAP-04. Red indicates higher source power in LAP than CONTROL, whereas blue indicates higher source power in CONTROL than LAP. Maps are displayed at FDR-corrected threshold p < .001 with a fixed t range from −4 to +4. This focused trial-level figure is distinct from the participant-level leave-one-out analysis of 819 unthresholded maps.

# Overall conclusion

The supplementary analyses indicate high overall stability of the EEG group-contrast topographies, together with condition-specific sensitivity concentrated mainly in Fear. Candidate-outlier and participant-level leave-one-out results should therefore be reported as robustness checks that qualify, rather than replace, the exploratory primary EEG findings.
